# The genome of a low‐seeded mandarin, Premier, displays major structural changes due to gamma irradiation

**DOI:** 10.1002/tpg2.70220

**Published:** 2026-03-19

**Authors:** Upuli Nakandala, Agnelo Furtado, Robert J. Henry

**Affiliations:** ^1^ Department of Molecular Biology and Biotechnology, Faculty of Science University of Peradeniya Peradeniya Sri Lanka; ^2^ ARC Centre of Excellence for Plant Success in Nature and Agriculture University of Queensland Brisbane Australia; ^3^ VinUni Bigdata Research Institute VinUniversity Hanoi Vietnam

## Abstract

The mandarin (*Citrus reticulata*) variety Premier (11C017) is a gamma‐irradiated mutant hybrid derived from a cross between Murcott and Ellendale. The variety Premier exhibits favorable traits, including good fruit size and productivity similar to its progenitor variety (01C011), along with a reduced seed count compared to the progenitor. Here, we developed haplotype‐resolved genomes for both Premier and its parental line Ellendale using PacBio HiFi and Hi‐C sequencing, followed by a combination of de novo and reference‐guided assembly approaches. The size of the assemblies ranged from 320 to 337 Mb, with N50s more than 31 Mb, and more than 98% BUSCO for the assembly and annotation. Comparative analysis revealed multiple structural rearrangements including inversions, translocations, and duplications in the Premier haplotypes relative to the parental genomes. Notably, we identified heterozygous reciprocal translocations (between Chr2 and Chr4 in haplotype 1, and Chr5 and Chr7 in haplotype 2) and a large heterozygous inversion (∼22 Mb on Chr2 of haplotype 1) as prominent rearrangements unique to Premier. These complex structural variants may disrupt normal meiotic pairing and gamete formation, potentially contributing to the observed reduction in seed number. These findings suggest that structural rearrangements may play a significant role in the reduction of the seed content of gamma‐irradiated plants.

## Plain Language Summary

Mandarin oranges are an important fruit crop, and seedless or low‐seed varieties are highly desirable for consumers. The variety Premier is a new type of mandarin developed from its parents Murcott and Ellendale and shows good fruit size and productivity but has fewer seeds. We analyzed the complete genetic makeup of Premier and its parents Ellendale and Murcott to understand why Premier has fewer seeds. Our study found that Premier's genome has unique structural changes, including rearrangements and inversions of large DNA segments, that are not present in its parents. These changes could interfere with normal cell division during reproduction, leading to fewer seeds in Premier fruits. These findings suggest that specific changes in the DNA structure may help create low‐seed or seedless fruit varieties, which could be useful for breeding better mandarins in the future.

**Abbreviations**
EL: Ellendale
hap: haplotype
MT: Murcott
Prem: Premier

## 1 INTRODUCTION

Gamma irradiation induces seedlessness in citrus through several biological mechanisms that abolish normal reproductive processes. The primary mechanism involves chromosomal aberrations and DNA damage in reproductive tissues, which results in meiotic irregularities during pollen and ovule development (Predieri, 2001). These disruptions result in the development of nonviable gametes or complete sterility due to improper chromosome pairing and segregation (Till et al., 2007). Additionally, gamma rays can induce physiological and biochemical changes in developing flower buds, affecting hormonal balance and disrupting the signaling pathways essential for fertilization and embryo development (Jan et al., 2012). In citrus, radiation‐induced mutations may also impair pollen tube growth and ovule viability, which in turn prevent successful fertilization even when viable pollen is present (Hearn, 1986). These combined effects ultimately result in the development of parthenocarpic fruit, where fruits develop without the formation of functional seeds.

Murcott (MT), a mandarin (*Citrus reticulata*) variety with some introgression from *C. reticulata* Blanco, has desirable traits; however, its high seed count (> 20 seeds per fruit) and late‐season maturity make it a suitable candidate for mutation breeding. The mandarin variety IrM2 was recently shown to have undergone major structural changes resulting from gamma irradiation of its parental variety, MT (Nakandala et al., 2025). These changes led to a reduced seed count, earlier development of red peel coloration, and fruit that was edible approximately 2 weeks earlier than the progenitor (Smith, 2006). Recently developed haplotyped‐resolved genomes of IrM2 revealed a range of haplotype‐specific structural rearrangements including translocations, inversions, duplications, and INDELs, suggesting their potential involvement in the expression of key traits (Nakandala et al., 2025).

**FIGURE 1:**
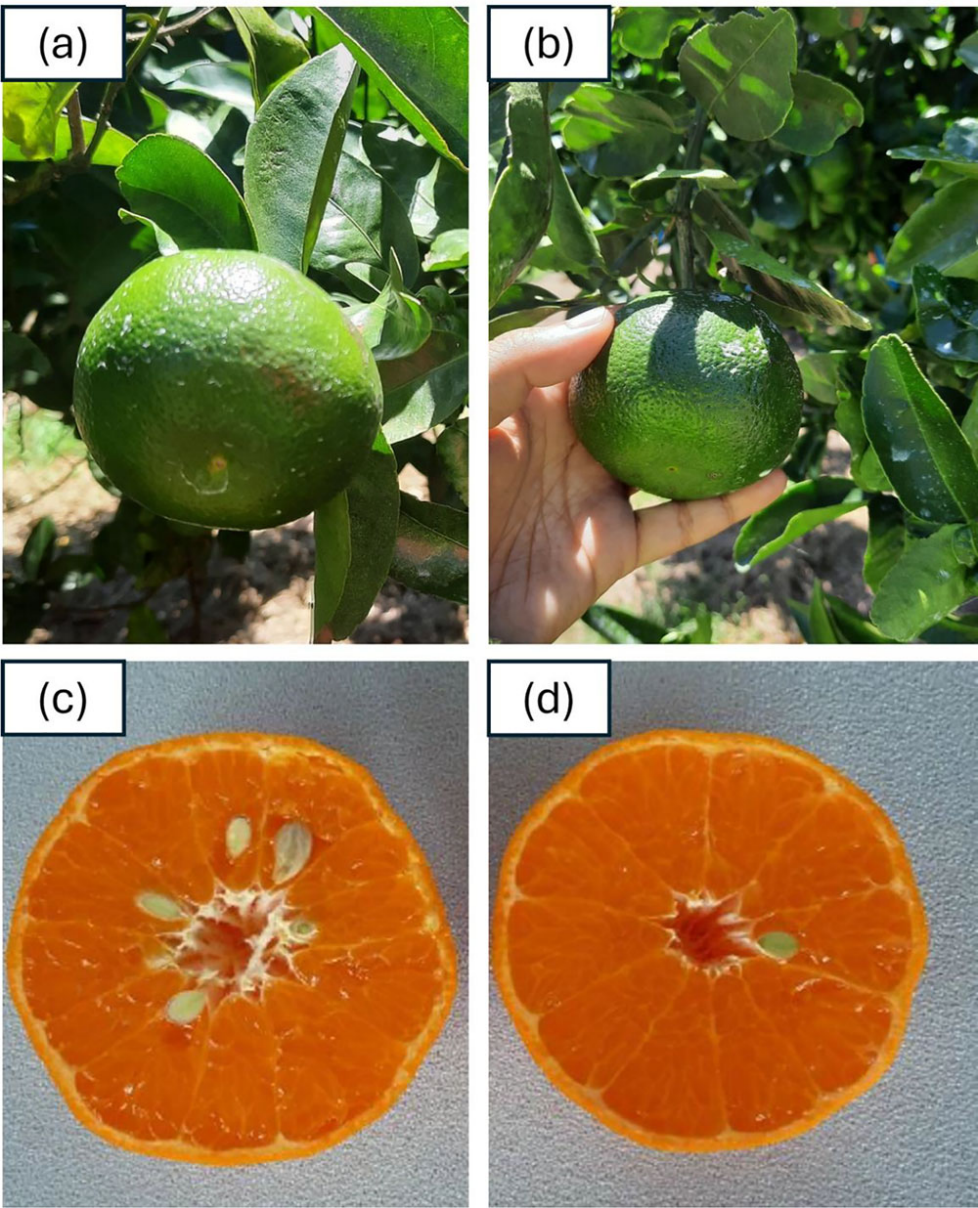
The two mandarin varieties: the hybrid variety (01C011), derived from sexual hybridization between Murcott and Ellendale, and the mutant variety Premier (11C017), derived from gamma irradiation of the hybrid 01C011. (a) The unripened fruit of the hybrid variety (01C011). (b) The unripened fruit of the mutant variety Premier (11C017). (c) The ripened fruit of the hybrid variety having more seeds. (d) The ripened fruit of the mutant variety having a smaller number of seeds. The images (c) and (d) were taken from the “Hort Innovation project report CT15017”.

The cultivar Ellendale (EL) produces large, seedy, and easy to peel fruits (Barry et al., 2020). The sexual hybridization between MT and EL mandarins produced a hybrid individual (01C011), with a desired combination of commercially important traits except with fruits that were very seedy (Figure 1a,c). The hybrid variety 01C011 was then subjected to mutation breeding, which created a population with a wide variation in seediness. A genotype from this population (11C017), with a reduced number of seeds compared to the progenitor, was given the commercial name Premier (Prem) (Figure 1b,d). Both Prem and IrM2 varieties express the common desirable trait of low seed content, which is particularly valued in citrus, as it enhances consumer appeal and marketability, particularly for fresh fruit consumption (Yin et al., 2023). Prem also had good fruit size and productivity. Here, we report the sequencing and assembly of the genomes of Prem and the parent EL to investigate the impact of gamma irradiation on the Prem genome.

## 2 MATERIALS AND METHODS

### 2.1 Sample collection, genome assembly, and annotation

Leaf tissue from young, immature leaves of Prem and EL cultivars was collected from the Department of Agriculture and Fisheries (DAF) arboretum in Bundaberg, Queensland, Australia. Total genomic DNA and RNA were extracted from pulverized tissue. PacBio HiFi sequencing and RNA sequencing were performed for both genomes as described by Nakandala et al. (2023). Hi‐C sequencing was conducted using an Arima Genomics method at Australian National University. Prior to assembly, Hi‐C reads were processed in CLC Genomic Workbench 24.0.1, which included quality and adapter trimming, followed by the removal of molecular barcodes. Contig‐level assemblies were generated from PacBio HiFi reads integrated with Hi‐C data using Hifiasm (v. 0.19.8) in Hi‐C mode with specified phasing parameters. Scaffolding for each haplotype was performed independently using a pipeline involving BWA aligner, Arima mapping (https://github.com/ArimaGenomics/mapping_pipeline), and SALSA scaffolding pipeline (Ghurye et al., 2017), as detailed in Nakandala et al. (2024).

The reference MT assemblies were developed as detailed in our previous research (Nakandala et al., 2025). To construct pseudochromosomes of the two new genomes (Prem and EL), first the scaffolds from the EL haplotype (EL‐hap) assemblies were independently aligned to the corresponding Murcott haplotype (MT‐hap) assemblies for chromosome assignment. For Prem, scaffolds were aligned to both the MT and EL assemblies. Scaffolds assigned to each chromosome were manually joined with intervening Ns, and their orientation was standardized according to the published *C. australis* A. Cunn. Ex Mundie genome (Nakandala et al., 2023) to maintain consistency across Australian lime genomes. For annotation, repeat elements were identified de novo using RepeatModeler2 (v. 2.0.1) (Flynn et al., 2020) and soft‐masked with RepeatMasker (v. 4.0.9_p2) (Chen, 2004). Quality‐trimmed RNA sequencing (RNA‐seq) reads were aligned to the masked genome using HISAT2 (Kim et al., 2019). Structural and functional gene annotation was performed according to our prior methodology, with gene prediction conducted using Braker3 (Hoff et al., 2019; Nakandala et al., 2024; BioBam, 2019). Telomeric regions in assemblies were detected through a combination of manual curation and computational analysis using tidk (telomere identification toolkit; https://github.com/tolkit/telomeric‐identifier).

Structural variations and syntenic relationships between homologous chromosomes of the parental genomes were examined using SyRI (Goel et al., 2019). Whole‐genome alignments were performed using nucmer (–maxmatch) with parameters ‐c 100 ‐b 500 ‐l 50 to capture all potential alignments between the two genomes. The resulting alignments were filtered using the delta‐filter tool. The filtered alignments were subsequently converted to a tab‐delimited coordinate format using the show‐coords command. For comprehensive identification and classification of structural genomic variants, the alignment files were processed using Syri with default parameters. Finally, the genomic structures and rearrangements predicted by Syri were visualized using plotsr. Additionally, each Premier haplotype (Prem‐hap) was compared to both MT‐hap and EL‐hap using SyRI to determine the parental origin of each chromosome of Prem‐haps.

### 2.2 Low‐complexity mapping analysis

To identify repetitive and unique genomic regions across Prem‐haps, k‐mer diversity analysis was performed using sliding windows (50 kb window size, 25 kb step size) across each chromosome. The complexity scores were calculated at k = 10 (distant memory, capturing long‐range repetitive elements) and k = 3 (local memory, capturing short‐range simple repeats). Complexity was measured as the ratio of unique k‐mers to total k‐mers within each window, with scores ranging from 0 (highly repetitive) to 1 (unique sequences). Analysis was performed using custom R scripts (available upon request) with Biostrings (v2.74.1) and ggplot2 (v4.0.1) packages (R Core Team, 2024).

### 2.3 Analysis of a nonplant content in Premier genome

In the Prem‐hap assemblies, several scaffolds could not be anchored to chromosomes based on alignment with parental genomes. However, these scaffolds were retained in the final assembly because their inclusion increased BUSCO scores, suggesting they contain important plant genes. Additionally, some small scaffolds possessed telomeric sequences at their termini, indicating they likely represent fragments of the nuclear genome.

To assess whether these unanchored scaffolds harbored nonplant content, all unanchored scaffolds from both haplotype assemblies were screened against the NCBI viral RefSeq representative genomes database using BLASTn with an e‐value threshold of ≤1e‐5. A positive control using tobacco mosaic virus (accession NC_001367.1) confirmed the analysis was functioning correctly and capable of detecting viral sequences when present.

## 3 RESULTS

The assemblies of EL‐hap were generated using 59 Gb of PacBio HiFi reads (∼161X from two PacBio SMRT cells) and 125 Gb of Hi‐C reads (∼382X). The same set of scaffolds of the two EL‐hap assemblies were aligned with MT‐hap assemblies (Figures S1 and S2), allowing them to be assigned to the corresponding chromosomes. EL‐hap1 assembly (EL‐hap1) had four pseudochromosomes with telomeres at both ends and the Ellendale hap2 (EL‐hap2) assembly had two pseudochromosomes with telomeres at both ends, representing full chromosomes. For all other pseudochromosomes, telomeres were present at only one end (Tables S1 and S2). The size of the nine pseudochromosomes was 316.2 Mb for hap1 assembly and 304.6 Mb for hap2 assembly. The size of the whole genome including the unplaced scaffolds was 326.3 Mb for the hap1 assembly and 334.4 Mb for the hap2 assembly.

**FIGURE 2:**
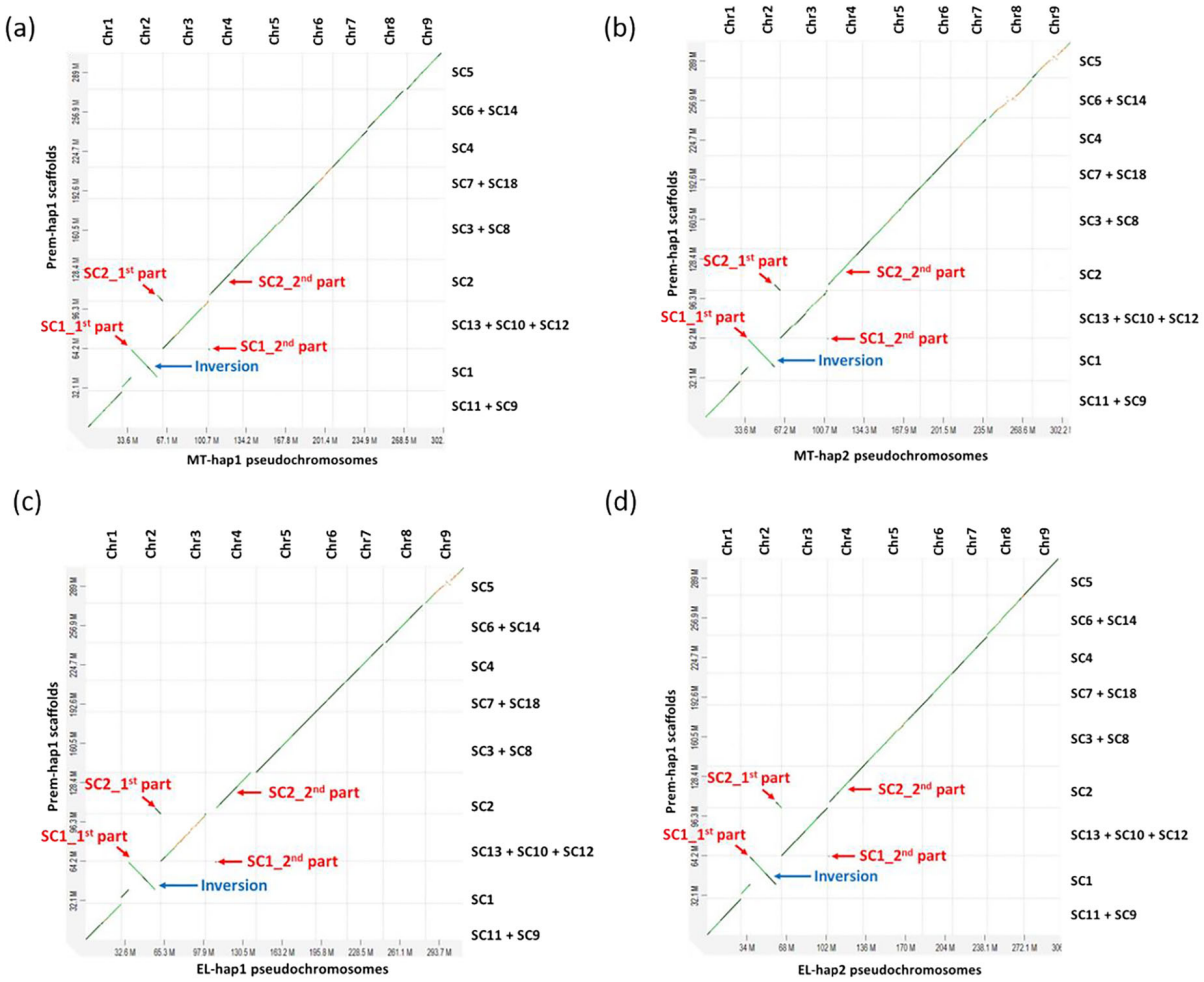
The alignment of Prem‐hap1 scaffolds against the MT and EL haplotype assemblies. The same set of Prem‐hap1 scaffolds corresponded to each chromosome of MT‐hap1 assembly (a), MT‐hap2 assembly (b), EL‐hap1 assembly (c), and EL‐hap2 assembly (d). A major part of the SC1 of Prem‐hap1 (SC1_1st part) corresponded to Chr2 of parental haplotypes. Another small part of the SC1 of Prem‐hap1 (SC1_2nd part) corresponded to Chr4 of parental haplotypes. An inversion was also found in SC1_1st part. A major part of the SC2 of Prem‐hap1 (SC2_2nd part) corresponded to Chr4 of parental haplotypes. Another small part of the SC2 of Prem‐hap1 (SC2_1st part) corresponded to Chr2 of parental haplotypes.

**FIGURE 3:**
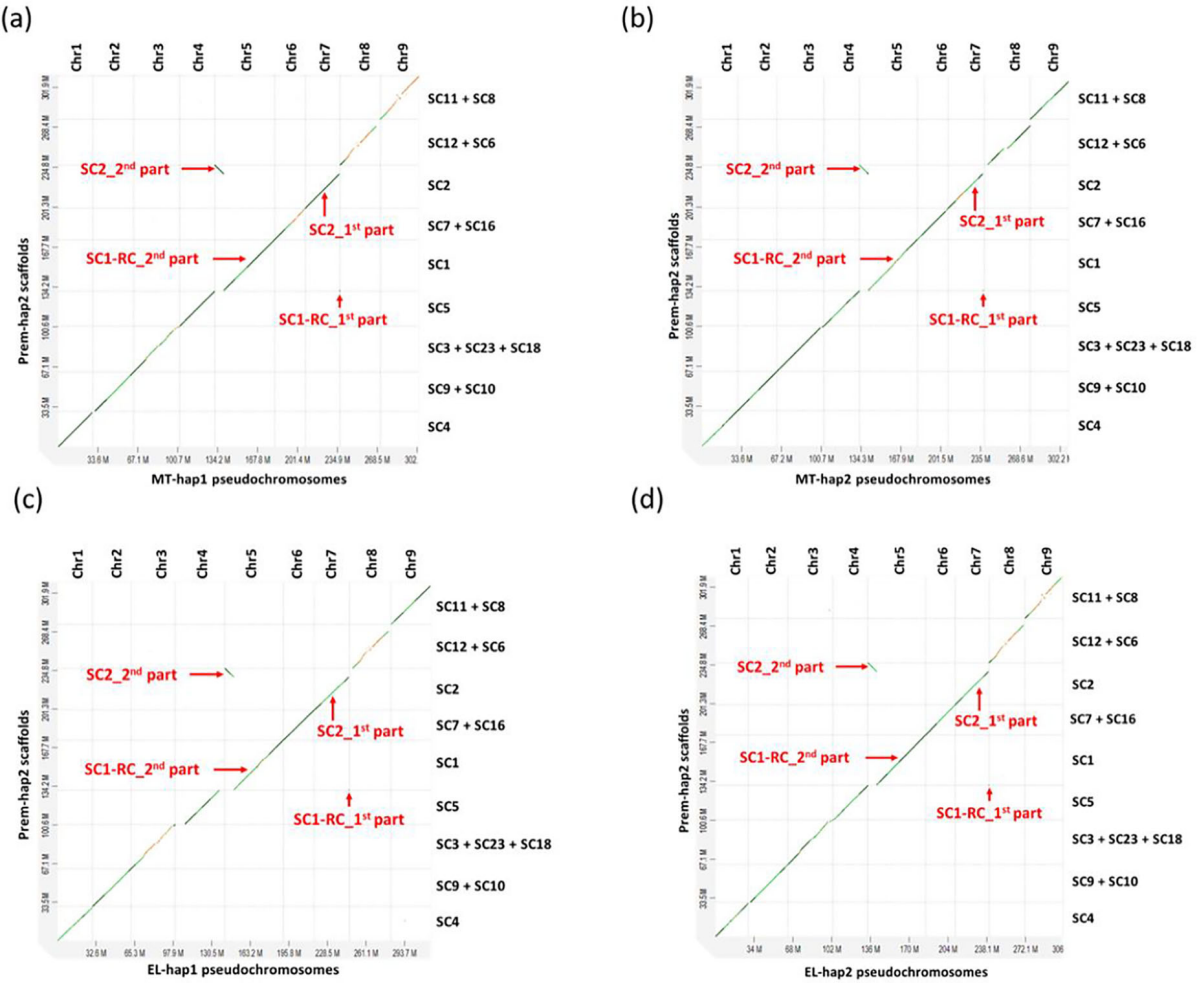
The alignment of Prem‐hap2 scaffolds against the MT and EL haplotype assemblies. The same set of Prem‐hap2 scaffolds corresponded to each chromosome of MT‐hap1 assembly (a), MT‐hap2 assembly (b), EL‐hap1 assembly (c), and EL‐hap2 assembly (d). A major part of the SC1 of Prem‐hap2 (SC1‐RC_2nd part) corresponded to Chr5 of the parental haplotypes. Another small part of the SC1 of Prem‐hap2 (SC1‐RC_1st part) corresponded to Chr7 of the parental haplotypes. A major part of the SC2 of Prem‐hap2 (SC2_1st part) corresponded to Chr7 of the parental haplotypes. Another small part of the SC2 of Prem‐hap2 (SC2_2nd part) corresponded to Chr5 of the parental haplotypes.

The assemblies of Prem‐hap were generated using 64 Gb of PacBio HiFi reads (∼174X from two PacBio SMRT cells) and 125 Gb of Hi‐C reads (∼382X). The scaffolds of Premier hap1 (Prem‐hap1) and Premier hap2 (Prem‐hap2), that were correspondent with MT‐hap and EL‐hap assemblies, were assigned to the corresponding chromosomes (Figures 2 and 3). In the Prem‐hap1 assembly, four pseudochromosomes had telomeres at both ends and five pseudochromosomes had telomeres at one end (Table S3). In Prem‐hap2 assembly, four pseudochromosomes had telomeres at both ends, four pseudochromosomes had telomeres at one end, and one pseudochromosome had no telomeres (Table S4). The size of the nine pseudochromosomes was 304.8 Mb for hap1 assembly and 312.7 Mb for hap2 assembly. The size of the whole genomes including the unplaced scaffolds was 320.9 Mb for hap1 assembly, while it was 336.8 Mb for hap2 assembly.

The assembly and the annotation statistics of the four whole genome assemblies (nine pseudochromosomes and unplaced scaffolds) are given in Table 1.

**TABLE 1:** The assembly and the annotation statistics of the whole genome assemblies of Ellendale and Premier varieties.

Name of the variety	Type of the assembly	Size (Mb)	Assembly BUSCO (%)	Assembly N50 (Mb)	No. of genes	No. of CDS	Annotation BUSCO (%)
Ellendale	hap1	326.3	98.1	35.2	28,024	32,717	98.6
	hap2	334.4	98.9	31.5	43,345	48,129	98.6
Premier	hap1	320.9	98.1	32.5	28,762	33,280	98.6
	hap2	336.8	98.9	36.4	38,795	43,640	98.8

### 3.1 The structural differences in Prem‐haps that likely resulted from irradiation

Any structural rearrangement in Prem haplotypes with respect to the four parental haplotype assemblies (MT and EL) was likely a mutation derived from irradiation. In contrast, the rearrangements in Prem haplotypes that were not present with respect to all the four parental haplotypes were attributed to the haplotype variations. One of the prominent rearrangements was the large inversion found in Chr2 in Prem‐hap1 with respect to the four parental assemblies (Figure S2). The inversion in Chr2 was heterozygous as it was found only in Prem‐hap1; however, it was absent in the Prem‐hap2 assembly. The other type of major rearrangement was the heterozygous, reciprocal translocations in the two haplotypes of Prem. A translocation was identified in the Prem‐hap1 assembly between the Chr2 and Chr4 (Figure 2), and another translocation was observed in the Prem‐hap2 assembly between the Chr5 and Chr7 (Figure 3).

### 3.2 The origin of Premier chromosomes from parents

**FIGURE 4:**
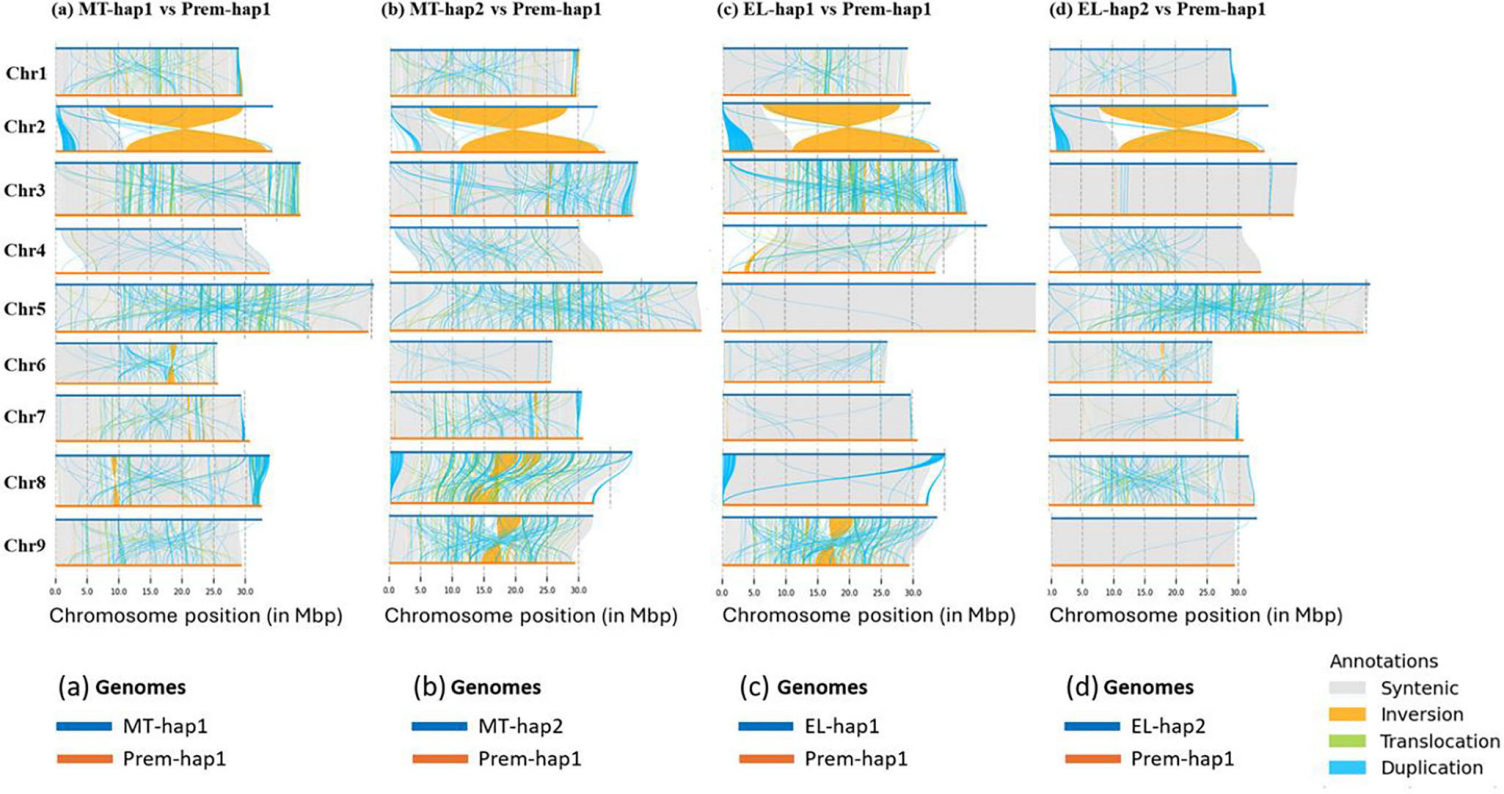
The synteny and structural variations plots between Prem‐hap1 and parental haplotype assemblies. All the chromosomes in Prem‐hap1 showed a relatively high synteny and less structural variations with respect to EL‐haps. For some chromosomes such as Chr3, Chr5, Chr8, and Chr9, the Prem‐hap1 chromosomes showed high synteny with one of the EL‐haps. Chr2 had a major inversion with respect to all parental haplotypes.

**FIGURE 5:**
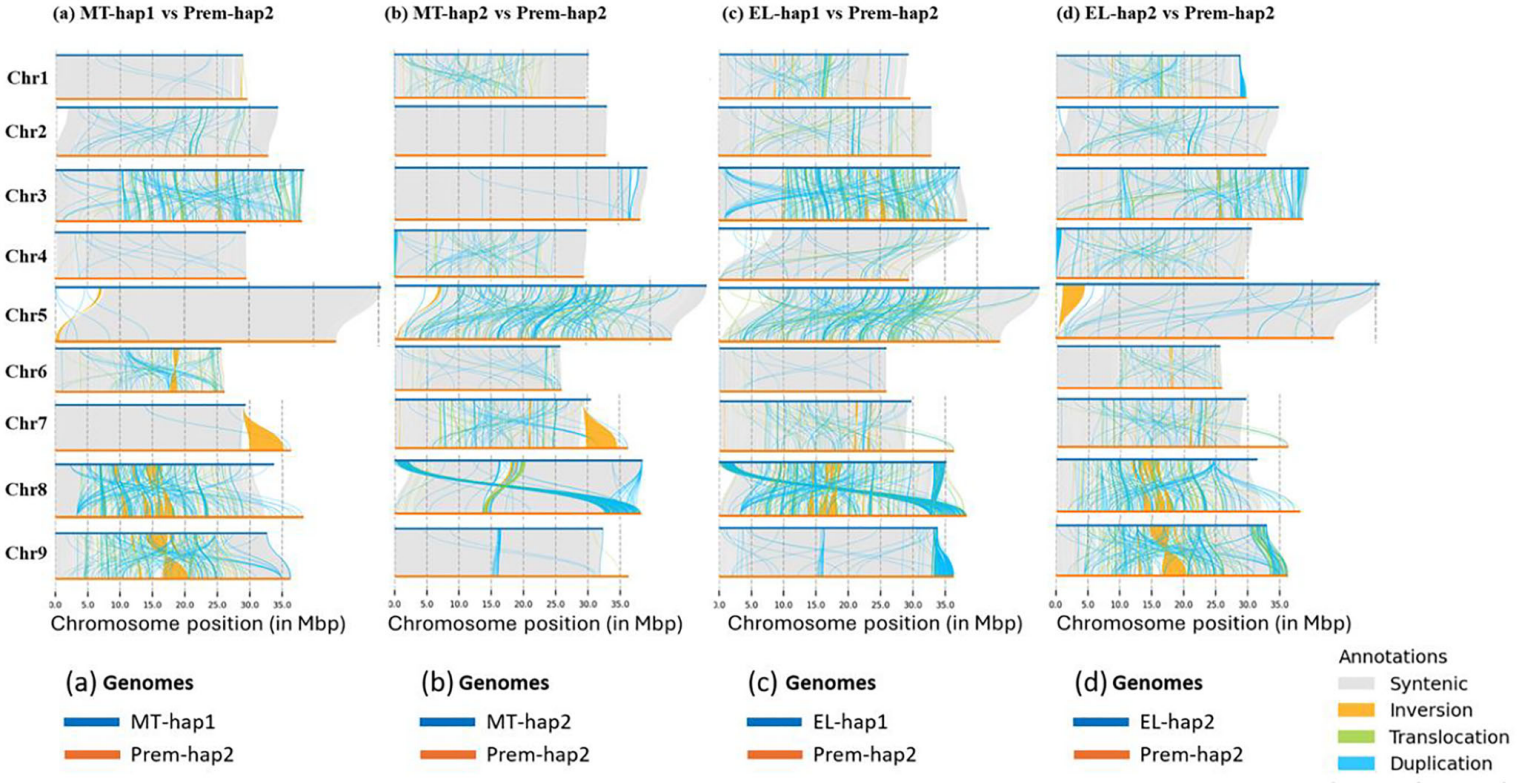
The synteny and structural variations plots between Prem‐hap2 and parental haplotype assemblies. All the chromosomes in Prem‐hap2 showed a relatively high synteny and fewer structural variations with respect to MT‐haps. These structural variations predominantly included inversions, translocations, and duplications. Inversions were prominent in Chr7, Chr8, and Chr9; translocations were prominent in Chr3, Chr5, Chr8, and Chr9, and duplications were prominent in Chr3, Chr5, Chr6, Chr7, Chr8, and Chr9.

The structural variations plots revealed high synteny and fewer structural differences between the chromosomes of the Prem‐hap1 assembly and EL‐hap assemblies (Figure 4), except for Chr6, where MT‐hap2 also showed high synteny with Prem‐hap1. In contrast, the structural variations plots showed high synteny and less structural variations between the chromosomes of the Prem‐hap2 assembly and MT‐hap assemblies (Figure 5), except for Chr6, where EL‐haps also showed high synteny with Prem‐hap2. These plots revealed that the chromosomes of Prem‐hap1 might have derived from EL, while those of Prem‐hap2 might have derived from MT (the origin of Chr6 is uncertain). The structural variations identified in Prem chromosomes with respect to their parental chromosomes could be due to recombination between the parental haplotypes (between MT‐hap1 and MT‐hap2, and between EL‐hap1 and EL‐hap2) and the subsequent irradiation.

The structural comparisons between parental and Prem‐hap assemblies revealed numerous structural variations in each comparison. Overall, duplications, translocations, and highly diverged regions represented the most abundant classes of structural variation in each comparison, contributing substantially to genomic coverage. In contrast, inversions, insertions, deletions, copy gains, copy losses, and tandem repeats occurred at lower frequencies and covered relatively small genomic regions (Tables 2 and 3, Tables S5–S12). Prem‐hap1 assembly showed a higher number and greater genomic coverage of variants relative to the parental MT‐hap2 compared to MT‐hap1. However, Prem‐hap2 assembly showed a higher number and greater genomic coverage of variants relative to the parental MT‐hap1 compared to MT‐hap2 (Table 2). In the EL–Prem comparison, both Prem‐hap1 and Prem‐hap2 assemblies showed higher numbers and greater genomic coverage of variants relative to the parental EL‐hap1 than to EL‐hap2, with the exception of tandem repeats, which were more abundant in Prem‐hap2 relative to EL‐hap2 (Table 3).

**TABLE 2:** Types, numbers, and genomic coverage of structural variations in MT‐Prem assembly comparisons.

Type of variation		MT‐hap1 (Ref) and Prem‐hap1 (Query)	MT‐hap1 (Ref) and Prem‐hap2 (Query)	MT‐hap2 (Ref) and Prem‐hap1 (Query)	MT‐hap2 (Ref) and Prem‐hap2 (Query)
Inversions_Query	Number	66	89	109	53
	Genomic coverage (Mb)	25.9	16.1	32.6	6.2
Translocations_Query	Number	1862	1966	2524	1239
	Genomic coverage (Mb)	9.8	9.5	12.2	5.7
Duplications_Ref	Number	8608	6830	8995	4539
	Genomic coverage (Mb)	24.8	19.3	31.2	17.2
Duplications_Query	Number	6118	6699	6574	3511
	Genomic coverage (Mb)	17.1	17.2	16.7	9.2
Insertions_Query	Number	704	683	1000	369
	Genomic coverage (Mb)	1.2	1.3	2.1	0.5
Deletions_Ref	Number	659	687	997	445
	Genomic coverage (Mb)	1.3	1.6	2.0	0.6
Copy gains_Query	Number	981	881	1334	569
	Genomic coverage (Mb)	2.7	2.5	3.7	5.5
Copy losses_Ref	Number	966	843	1365	558
	Genomic coverage (Mb)	2.5	2.3	4.4	1.4
Highly diverged regions_Query	Number	2049	2299	2949	1035
	Genomic coverage (Mb)	5.0	14.5	8.9	2.6
Tandem repeats_Query	Number	186	98	187	154
	Genomic coverage (Mb)	0.8	0.5	0.7	1

Abbreviations: Query: the query genome; Ref: reference genome; MT: Murcott; Prem: Premier.

**TABLE 3:** Types, numbers, and genomic coverage of structural variations in EL‐Prem assembly comparisons.

Type of variation		EL‐hap1 (Ref) and Prem‐hap1 (Query)	EL‐hap1 (Ref) and Prem‐hap2 (Query)	EL‐hap2 (Ref) and Prem‐hap1 (Query)	EL‐hap2 (Ref) and Prem‐hap2 (Query)
Inversions_Query	Number	83	96	39	83
	Genomic coverage (Mb)	28.4	6.6	23.1	9.8
Translocations_Query	Number	1872	2588	872	2114
	Genomic coverage (Mb)	8.2	12.9	4.5	9.8
Duplications_Ref	Number	5956	9218	4864	7258
	Genomic coverage (Mb)	24.1	32.5	11.7	25.9
Duplications_Query	Number	4631	7835	3403	5808
	Genomic coverage (Mb)	13.0	19.5	11.9	15.6
Insertions_Query	Number	683	789	333	717
	Genomic coverage (Mb)	1.2	1.4	0.5	1.1
Deletions_Ref	Number	728	913	295	723
	Genomic coverage (Mb)	1.1	1.4	0.4	1.5
Copy gains_Query	Number	932	1071	408	979
	Genomic coverage (Mb)	2.4	2.7	0.9	3.1
Copy losses_Ref	Number	858	1059	431	967
	Genomic coverage (Mb)	2.2	2.6	1.1	2.3
Highly diverged regions_Query	Number	2552	2968	779	2065
	Genomic coverage (Mb)	7.2	7.4	1.4	7.1
Tandem repeats_Query	Number	82	136	102	208
	Genomic coverage (Mb)	0.4	0.5	0.7	1

Abbreviations: Query: the query genome; Ref: Reference genome; EL: Ellendale; Prem: Premier.

### 3.3 Low‐complexity mapping results

To identify repetitive elements and potential drivers of structural variation in Prem‐hap assemblies, we performed low‐complexity mapping across all nine chromosomes using k‐mer diversity analysis at distant (*k* = 10) and local (*k* = 3) memory scales (Figures S3–S20). Complexity scores represent the diversity of k‐mer sequences within sliding windows, where low scores indicate repetitive DNA (same sequences repeated) and high scores indicate unique sequences (diverse patterns). Distant memory maps revealed heterogeneous repetitive content distribution, with Chr2, Chr 8, and Chr9 displaying extensive terminal repetitive blocks (complexity scores near 0, spanning ≥5 Mb) likely representing telomeric and subtelomeric heterochromatin, while other chromosomes showed scattered low‐complexity regions indicative of distributed transposable element insertions. Haplotype comparison identified both conserved repetitive features and numerous haplotype‐specific low‐complexity regions. Notably, chromosome 5 displayed the highest overall repetitive content. The most dramatic haplotype divergence could be observed in Chr2, Chr8, and Chr9, where the hap2 harbored a substantially larger terminal repetitive block than hap1 in Chr8 and Chr9, while the reverse was true in Chr2. Local memory maps were largely uniform across the genome, indicating consistent short‐range sequence composition, with occasional spikes suggesting regions enriched in specific nucleotide motifs.

### 3.4 Non‐plant content in unanchored scaffolds

In the Prem‐hap1 assembly, nonchromosomal scaffolds (16.8 Mb total) showed no detectable viral sequences, including the citrus‐associated viruses CTV (citrus tristeza virus), CMBV (citrus yellow mosaic badnavirus), CitPRV (citrus psorosis‐related virus), and CCDaV (citrus chlorotic dwarf‐associated virus), indicating these sequences are free from known viral contamination. In contrast, the Prem‐hap2 assembly revealed viral sequence similarities in three of eight unanchored scaffolds (Scaffolds 14, 15, and 21). The identified sequences comprised bacterial phages and endogenous viral elements (EVEs) with varying sequence identities and alignment lengths (Table S13). Scaffold 14 contained sequences with low similarity (72%–81%) to bacterial and archaeal phages, Scaffolds 15 and 21 exhibited multiple hits to three viral groups: (i) citrus endogenous pararetrovirus (CitEPRV; 80%–88% identity, 65–5049 bp alignments), (ii) choristoneura fumiferana granulovirus (89%–90% identity, 95 bp alignments), and (iii) pepper chlorotic spot virus (91.5% identity, 65–71 bp alignments). Importantly, no exogenous citrus‐pathogenic viruses (CTV, CMBV, CitPRV, and CCDaV) were detected in any scaffold, confirming the assembly is free from known citrus viral pathogens.

## 4 DISCUSSION

A low seed content is a valuable trait in many fruit crops including grape (di Rienzo et al., 2021), banana (Backiyarani et al., 2021), and date palm (Hamza et al., 2016). In citrus, low seed content has also gained significant importance due to its contribution to easier consumption and improved processing efficiency (Yin et al., 2023). The variety Premier is a mutant derived from a hybrid individual that originated from sexual hybridization between the two parental varieties MT and EL. The determination of the chromosomes of Prem‐haps from their parents was important to understand the likely origin of their structural variations. The structural rearrangements observed in the Prem‐hap assemblies may be attributed to three possible causes. They could be due to pre‐existing structural polymorphisms between parental haplotypes, sexual recombination between parental haplotypes, and irradiation, which made it difficult to make definitive conclusions about their origins. The rearrangements relative to both the parental haplotypes were likely derived from irradiation. However, the rearrangements in Prem relative to any one of the parental haplotypes was more likely a consequence of recombinations between the parental haplotypes or the differences in the structure of the two parental haplotypes (MT‐hap1 and MT‐hap2 or EL‐hap1 and EL‐hap2).

Structural rearrangements induced by irradiation have been previously investigated in citrus and other fruit crops using chromosome‐level genome assemblies. A range of mutations including deletions, insertions, translocations, tandem duplications, and inversions have been identified in radiation‐induced mutants of sweet orange (Wu et al., 2023) and the mandarin variety IrM2 (Nakandala et al., 2025). In grape, heterozygous inversions have been linked to low seed numbers (Wang et al., 2024), while both inversions and translocations were detected in seedless mutants compared to seeded genomes of date plum (Mao et al., 2021). The mutant cultivars developed through irradiation from seedy, self‐incompatible, and parthenocarpic varieties such as Moncada and W. Murcott mandarins have been observed to produce fewer seeds, even under cross‐pollination conditions. This reduction in seed number is primarily linked to decreased viability of both pollen and ovules, resulting in male and female sterility (Bermejo et al., 2011; Zhu, 2018). In particular, the gametic sterility observed in the W. Murcott mutant Tango was studied using short read sequencing and attributed to complex structural mutations including heterozygous translocations, inversions, and deletions—induced by irradiation (Zhu, 2018). Heterozygous translocations have the potential to cause meiotic nondisjunction by disrupting normal chromosome pairing during meiosis. When such genetically unbalanced gametes are fertilized by normal gametes, the resulting zygotes may carry chromosomal imbalances, often leading to nonviable embryos that are subsequently aborted (Forejt, 2001). Similarly, chromosomal inversions can affect reproductive success to varying degrees. While some inversions are likely to be associated with embryo developmental failure or increased rates of abortion, others may have minimal or no impact on fertility (Mennuti, 2019).

The common phenotypic feature induced by mutagenesis in the two mandarin varieties Premier and IRM2 was the low seed content. This study of the de novo assembled genome of Prem confirmed the presence of major chromosomal rearrangements in the gamma‐irradiated genome of this low‐seeded mandarin (heterozygous translocations and inversions) as we recently reported for another low‐seeded mandarin (Nakandala et al., 2025). As evidenced by previous studies, the presence of complex structural variants may interfere with normal meiotic chromosome pairing and gametogenesis, potentially leading to the reduced seed number observed in premier. The structural rearrangements identified in this study based on genome assemblies should be further validated using complementary approaches such as polymerase chain reaction, fluorescence in situ hybridization, and RNA‐seq to confirm their presence in Prem. Although additional functional validation is needed, our results indicate that structural rearrangements particularly heterozygous translocations and inversions could be a major factor contributing to the reduced number of seeds seen in gamma‐irradiated citrus mutants such as Prem. Upon validation, gene‐editing techniques—such as CRISPR/Cas9—could be employed to introduce targeted structural changes in the genomes of citrus varieties using haplotype‐specific guide RNAs (gRNAs) and donor templates for homology‐directed repair (HDR). This strategy may facilitate the induction of low seed numbers in citrus without causing deleterious genomic effects typically associated with irradiation.

The haplotype‐specific repetitive elements identified through low‐complexity mapping provide mechanistic insights into structural and expression divergence between haplotypes. Chr5, with the highest repetitive content, and Chr2, Chr8, and Chr9, with the most dramatic haplotype differences, likely represent hotspots for recent transposon activity that may drive the structural variants we observed. The overlap between low‐complexity regions and structural variant hotspots suggests transposable elements act as both direct sources of variation and facilitators of chromosomal rearrangements. Additionally, proximity of repetitive elements to differentially expressed genes raises the possibility of TE‐mediated gene regulation through epigenetic modifications or regulatory element disruption (Galindo‐González et al., 2018). The distinct repetitive landscapes between haplotypes may thus contribute to both structural genome divergence and haplotype‐specific expression patterns. These findings underscore the importance of haplotype‐resolved assembly for characterizing repetitive content and understanding its functional consequences in heterozygous citrus genomes.

The investigation of small nonchromosomal scaffolds revealed the presence of bacterial/archaeal viruses with low similarity (72%–81%) in scaffold 14, which are likely false positives from repetitive sequences. Plant genomes are heavily composed of repetitive DNA elements (3%–80%). These repetitive, noncoding, or transposable elements can generate “false positives” in homology‐based searches (e.g., BLASTn) by appearing superficially similar to bacterial phage or archaeal virus genomes. They are also likely derived from contamination from bacteria associated with the source tissue during DNA extraction (Koskella & Taylor, 2015)—a common occurrence in plant genome sequencing. Scaffolds 15 and 21 had a mixture of sequences showing similarity with CitEPRV, choristoneura fumiferana granulovirus, and pepper chlorotic spot virus. Endogenous pararetroviruses (EPRVs) are a class of EVEs that were detected as expected, and consistent with published reports documenting hundreds to thousands of EPRV segments across all Citrinae genomes (De Tomas & Vicient, 2022; Yu et al., 2019). Choristoneura fumiferana granulovirus is an insect baculovirus also reported as an endogenous element in strawberry plant genomes (Wang et al., 2024), suggesting ancient horizontal transfer events common across plant lineages. These EVEs represent ancient integrations dating to millions of years ago and are a normal component of citrus genome architecture, potentially contributing to antiviral defense mechanisms. Pepper chlorotic spot virus (91% identity, 71 bp) may represent conserved plant defense response elements or could be a false positive from short, conserved sequences rather than viral integration. Importantly, no plant‐pathogenic viruses or the specific citrus viruses of concern (CTV, CMBV, CitPRV, and CCDaV) were detected, indicating the assembly is free from known citrus viral pathogens.

## AUTHOR CONTRIBUTIONS

**Upuli Nakandala**: Conceptualization; data curation; formal analysis; investigation; methodology; validation; visualization; writing—original draft. **Agnelo Furtado**: Conceptualization; methodology; project administration; supervision; writing—review and editing. **Robert J. Henry**: Conceptualization; funding acquisition; methodology; project administration; supervision; writing—review and editing.

## CONFLICT OF INTEREST STATEMENT

The authors declare no conflicts of interest.

## Supporting information




**Supplemental Figure S1** The alignment of the scaffolds of EL‐hap1 assembly against the MT‐haps.

**Supplemental Figure S2** The alignment of the scaffolds of Ellendale hap2 assembly against the Murcott haplotypes.

**Supplemental Figure S3** Low‐complexity maps of two haplotype assemblies of Citrus Chr1 using distant (k = 10) memory analysis, revealing long‐range sequence patterns.

**Supplemental Figure S4** Low‐complexity maps of two haplotype assemblies of Citrus Chr1 using local (k = 3) memory analysis, revealing short‐range sequence patterns.

**Supplemental Figure S5** Low‐complexity maps of two haplotype assemblies of Citrus Chr2 using distant (k = 10) memory analysis, revealing long‐range sequence patterns

**Supplemental Figure S6** Low‐complexity maps of two haplotype assemblies of Citrus Chr2 using local (k = 3) memory analysis, revealing short‐range sequence patterns

**Supplemental Figure S7** Low‐complexity maps of two haplotype assemblies of Citrus Chr3 using distant (k = 10) memory analysis, revealing long‐range sequence patterns

**Supplemental Figure S8** Low‐complexity maps of two haplotype assemblies of Citrus Chr3 using local (k = 3) memory analysis, revealing short‐range sequence patterns

**Supplemental Figure S9** Low‐complexity maps of two haplotype assemblies of Citrus Chr4 using distant (k = 10) memory analysis, revealing long‐range sequence patterns

**Supplemental Figure S10** Low‐complexity maps of two haplotype assemblies of Citrus Chr4 using local (k = 3) memory analysis, revealing short‐range sequence patterns

**Supplemental Figure S11** Low‐complexity maps of two haplotype assemblies of Citrus Chr5 using distant (k = 10) memory analysis, revealing long‐range sequence patterns

**Supplemental Figure S12** Low‐complexity maps of two haplotype assemblies of Citrus Chr5 using local (k = 3) memory analysis, revealing short‐range sequence patterns

**Supplemental Figure S13** Low‐complexity maps of two haplotype assemblies of Citrus Chr6 using distant (k = 10) memory analysis, revealing long‐range sequence patterns

**Supplemental Figure S14** Low‐complexity maps of two haplotype assemblies of Citrus Chr6 using local (k = 3) memory analysis, revealing short‐range sequence patterns

**Supplemental Figure S15** Low‐complexity maps of two haplotype assemblies of Citrus Chr7 using distant (k = 10) memory analysis, revealing long‐range sequence patterns

**Supplemental Figure S16** Low‐complexity maps of two haplotype assemblies of Citrus Chr7 using local (k = 3) memory analysis, revealing short‐range sequence patterns

**Supplemental Figure S17** Low‐complexity maps of two haplotype assemblies of Citrus Chr8 using distant (k = 10) memory analysis, revealing long‐range sequence patterns

**Supplemental Figure S18** Low‐complexity maps of two haplotype assemblies of Citrus Chr8 using local (k = 3) memory analysis, revealing short‐range sequence patterns

**Supplemental Figure S19** Low‐complexity maps of two haplotype assemblies of Citrus Chr9 using distant (k = 10) memory analysis, revealing long‐range sequence patterns

**Supplemental Figure S20** Low‐complexity maps of two haplotype assemblies of Citrus Chr9 using local (k = 3) memory analysis, revealing short‐range sequence patterns

**Supplemental Table 1** EL‐hap1 scaffolds corresponding to MT‐hap1 and hap2 chromosomes
**Supplemental Table 2** EL‐hap2 scaffolds corresponding to MT‐hap1 and hap2 chromosomes
**Supplemental Table 3** Prem‐hap1 scaffolds corresponding to MT and EL haplotype chromosomes
**Supplemental Table 4** Prem‐hap2 scaffolds corresponding to MT and EL haplotype chromosomes
**Supplemental Table S5** Structural variants in Prem‐hap1 genome with respect to MT‐hap1
**Supplemental Table S6** Structural variants in Prem‐hap1 genome with respect to MT‐hap2
**Supplemental Table S7** Structural variants in Prem‐hap2 genome with respect to MT‐hap1

**Supplemental Table S8** Structural variants in Prem‐hap2 genome with respect to MT‐hap2
**Supplemental Table S9** Structural variants in Prem‐hap1 genome with respect to EL‐hap1

**Supplemental Table S10** Structural variants in Prem‐hap1 genome with respect to EL‐hap2

**Supplemental Table S11** Structural variants in Prem‐hap2 genome with respect to EL‐hap1

**Supplemental Table S12** Structural variants in Prem‐hap2 genome with respect to EL‐hap2
**Supplemental Table S13** The non‐plant content of small‐sized non‐chromosomal scaffolds in Prem‐hap2 assembly

## Data Availability

The whole genome sequence data and annotation data for EL and Prem hap assemblies have been submitted to Citrus genome database, under the accession numbers CGD25001 (Ellendale) and CGD25002 (Premier) (https://www.citrusgenomedb.org/).

## References

[tpg270220-bib-0001] Backiyarani, S. , Sasikala, R. , Sharmiladevi, S. , & Uma, S. (2021). Decoding the molecular mechanism of parthenocarpy in Musa spp. through protein–protein interaction network. Scientific Reports, 11(1), 14592. 10.1038/s41598-021-93661-3 34272422 PMC8285514

[tpg270220-bib-0002] Barry, G. H. , Caruso, M. , & Gmitter, F. G. Jr. (2020). Commercial scion varieties. In M. Talon , M. Caruso , & Fred G. G. Jr. (Eds.), The genus citrus (pp. 83–104). Elsevier.

[tpg270220-bib-0003] Bermejo, A. , Pardo, J. , & Cano, A. (2011). Influence of gamma irradiation on seedless citrus production: Pollen germination and fruit quality. Food Science & Nutrition, 2, 169–180.

[tpg270220-bib-0004] BioBam (2019). OmicsBox – Bioinformatics made easy (version 2.2.4) [Computer software]. BioBam Bioinformatics.

[tpg270220-bib-0005] Chen, N. (2004). Using repeat masker to identify repetitive elements in genomic sequences. Current Protocols in Bioinformatics, 5, 4.10.1–4.10.14. 10.1002/0471250953.bi0410s05 18428725

[tpg270220-bib-0006] De Tomás, C. , & Vicient, C. M. (2022). Genome‐wide identification of reverse transcriptase domains of recently inserted endogenous plant pararetrovirus (Caulimoviridae). Frontiers in Plant Science, 13, 1011565. 10.3389/fpls.2022.1011565 36589050 PMC9794742

[tpg270220-bib-0007] di Rienzo, V. , Imanifard, Z. , Mascio, I. , Gasser, C. S. , Skinner, D. J. , Pierri, C. L. , Marini, M. , Fanelli, V. , Sabetta, W. , Montemurro, C. , & Bellin, D. (2021). Functional conservation of the grapevine candidate gene INNER NO OUTER for ovule development and seed formation. Horticulture Research, 8, Article 29. 33518713 10.1038/s41438-021-00467-5 PMC7848007

[tpg270220-bib-0008] Flynn, J. M. , Hubley, R. , Goubert, C. , Rosen, J. , Clark, A. G. , Feschotte, C. , & Smit, A. F. (2020). RepeatModeler2 for automated genomic discovery of transposable element families. Proceedings of the National Academy of Sciences, 117, 9451–9457. 10.1073/pnas.1921046117 PMC7196820 32300014

[tpg270220-bib-0009] Forejt, J. (2001). Nondisjunction. In S. Brenner & J. H. Miller (Eds.), Encyclopedia of genetics (pp. 1345–1347). Academic Press.

[tpg270220-bib-0010] Galindo‐González, L. , Sarmiento, F. , & Quimbaya, M. A. (2018). Shaping plant adaptability, genome structure and gene expression through transposable element epigenetic control: Focus on methylation. Agronomy, 8(9), 180. 10.3390/agronomy8090180

[tpg270220-bib-0011] Ghurye, J. , Pop, M. , Koren, S. , Bickhart, D. , & Chin, C. S. (2017). Scaffolding of long read assemblies using long range contact information. BMC Genomics, 18(1), 527. 10.1186/s12864-017-3879-z 28701198 PMC5508778

[tpg270220-bib-0012] Goel, M. , Sun, H. , Jiao, W.‐B. , & Schneeberger, K. (2019). SyRI: Finding genomic rearrangements and local sequence differences from whole‐genome assemblies. Genome Biology, 20(1), 1–13. 10.1186/s13059-019-1911-0 31842948 PMC6913012

[tpg270220-bib-0013] Hamza, H. , Mrabet, A. , & Jiménez‐Araujo, A. (2016). Date palm parthenocarpic fruits (*Phoenix dactylifera* L.) cv. Deglet Nour: Chemical characterization, functional properties and antioxidant capacity in comparison with seeded fruits. Scientia Horticulturae Journal, 211, 352–357. 10.1016/j.scienta.2016.09.031

[tpg270220-bib-0014] Hearn, C. J. (1986). Development of seedless grapefruit cultivars through budwood irradiation. Journal of the American Society for Horticultural Science, 111(2), 304–306. 10.21273/JASHS.111.2.304

[tpg270220-bib-0015] Hoff, K. J. , Lomsadze, A. , and Borodovsky, M. , & Stanke, M. (2019). Whole‐genome annotation with BRAKER. Methods in Molecular Biology, 1962, 65–95. 31020555 10.1007/978-1-4939-9173-0_5 PMC6635606

[tpg270220-bib-0016] Jan, S. , Parween, T. , Siddiqi, T. O. , & Mahmooduzzafar (2012). Effect of gamma radiation on morphological, biochemical, and physiological aspects of plants and plant products. Environmental Reviews, 20(1), 17–39. 10.1139/a11-021

[tpg270220-bib-0017] Kim, D. , Paggi, J. M. , Park, C. , Bennett, C. , & Salzberg, S. L. (2019). Graph‐based genome alignment and genotyping with HISAT2 and HISAT‐genotype. Nature Biotechnology, 37, 907–915. 10.1038/s41587-019-0201-4 PMC7605509 31375807

[tpg270220-bib-0018] Koskella, B. , & Taylor, T. B. (2015). The potential role of bacteriophages in shaping plant‐bacterial interactions. In Bacteria‐plant interactions: Advanced research and future trends (pp. 199–220). Caister Academic Press. 10.21775/9781908230584

[tpg270220-bib-0019] Mao, W. , Yao, G. , Wang, S. , Zhou, L. , Chen, G. , Dong, N. , & Hu, G. (2021). Chromosome‐level genomes of seeded and seedless date plum based on third‐generation DNA sequencing and Hi‐C analysis. Forestry Research, 1, 9. 10.48130/FR-2021-0009 39524504 PMC11524226

[tpg270220-bib-0020] Mennuti, M. (2019). Cytogenetics: Part 2, Structural Chromosome Rearrangements and Reproductive Impact. In M. Norton , J. Kuller , & L. Dugoff (Eds.), Perinatal genetics (pp. 39–43). Elsevier.

[tpg270220-bib-0021] Nakandala, U. , Furtado, A. , Kharabian Masouleh, A. , Smith, M. W. , Mason, P. , & Henry, R. J. (2025). Characterizing the structural variations in the genome of the mandarin variety, IrM2, induced by gamma irradiation. Plant Biotechnology Journal, 23(9), 3814–3823. 10.1111/pbi.70205 40517402 PMC12392971

[tpg270220-bib-0022] Nakandala, U. , Furtado, A. , Masouleh, A. K. , Smith, M. W. , Williams, D. C. , & Henry, R. J. (2024). The genome of *Citrus australasica* reveals disease resistance and other species specific genes. BMC Plant Biology, 24, 260. 10.1186/s12870-024-04988-8 38594608 PMC11005238

[tpg270220-bib-0023] Nakandala, U. , Masouleh, A. K. , Smith, M. W. , Furtado, A. , Mason, P. , Constantin, L. , & Henry, R. J. (2023). Haplotype resolved chromosome level genome assembly of Citrus australis reveals disease resistance and other citrus specific genes. Horticulture Research, 10, uhad058. 10.1093/hr/uhad058 37213680 PMC10199705

[tpg270220-bib-0024] Predieri, S. (2001). Mutation induction and tissue culture in improving fruits. Plant Cell, Tissue and Organ Culture, 64(2–3), 185–210. 10.1023/A:1010623203554

[tpg270220-bib-0025] R Core Team . (2024). R: A language and environment for statistical computing. R Foundation for Statistical Computing. https://www.R‐project.org/

[tpg270220-bib-0026] Smith, M. W. (2006). Variety ‘IrM2’. Plant Varieties Journal, 19(3), 260–264. www.ipaustralia.gov.au/tools‐and‐research/professional‐resources/ip‐rights‐journals/plant‐varieties‐journals

[tpg270220-bib-0027] Till, B. J. , Cooper, J. , Tai, T. H. , Colowit, P. , Greene, E. A. , Henikoff, S. , & Comai, L. (2007). Discovery of chemically induced mutations in rice by TILLING. BMC Plant Biology, 7(1), 19. 10.1186/1471-2229-7-19 17428339 PMC1858691

[tpg270220-bib-0028] Wang, X. , Liu, Z. , Zhang, F. , Xiao, H. , Cao, S. , Xue, H. , Liu, W. , Su, Y. , Liu, Z. , Zhong, H. , Zhang, F. , Ahmad, B. , Long, Q. , Zhang, Y. , Liu, Y. , Gan, Y. , Hou, T. , Jin, Z. , Wu, X. , … Zhou, Y. (2024). Integrative genomics reveals the polygenic basis of seedlessness in grapevine. Current Biology, 34(16), 3763–3777. 10.1016/j.cub.2024.07.022 39094571

[tpg270220-bib-0029] Wu, B. , Yu, Q. , Deng, Z. , Duan, Y. , Luo, F. , & Gmitter, F. Jr. (2023). A chromosome‐level phased genome enabling allele‐level studies in sweet orange: A case study on citrus Huanglongbing tolerance. Horticulture Research, 10(1), uhac247. 10.1093/hr/uhac247 36643761 PMC9832951

[tpg270220-bib-0030] Yin, P. , Ding, W. , Zhang, H. , Liu, X. , Zhang, H. , Zeng, J. , & Xu, J. (2023). Morphological, physiological and molecular characteristics of the seedless ‘Hongjiangcheng’sweet orange. Horticultural Plant Journal, 9(3), 437–449. 10.1016/j.hpj.2022.10.001

[tpg270220-bib-0031] Yu, H. , Wang, X. , Lu, Z. , Xu, Y. , Deng, X. , & Xu, Q. (2019). Endogenous pararetrovirus sequences are widely present in Citrinae genomes. Virus Research, 262, 48–53. 10.1016/j.virusres.2018.05.018 29792903

[tpg270220-bib-0032] Zhu, Y. (2018). Genomic differences between W. Murcott Mandarin and its mutational derivative Tango. UC Riverside.

