# Supplementary figures and images for: The genome of a low‐seeded mandarin, Premier, displays major structural changes due to gamma irradiation
Source: Plant Genome. 2026 Mar 19;19(1):e70220. doi: 10.1002/tpg2.70220 (PMC13003167; doi:10.1002/tpg2.70220)

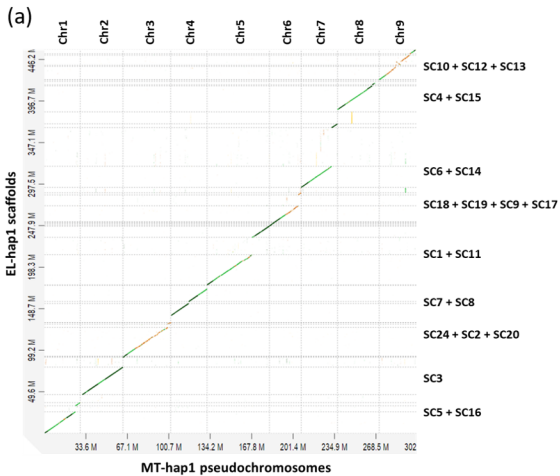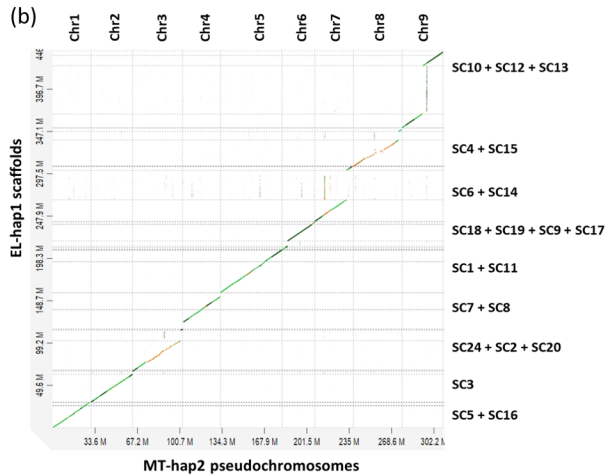

Supplement: Supplementary file 1 — Supplemental Figure S1 The alignment of the scaffolds of EL‐hap1 assembly against the MT‐haps. [file TPG2-19-e70220-s008.pdf]

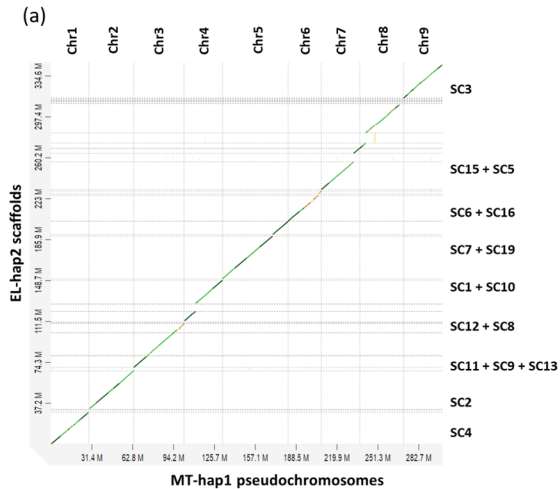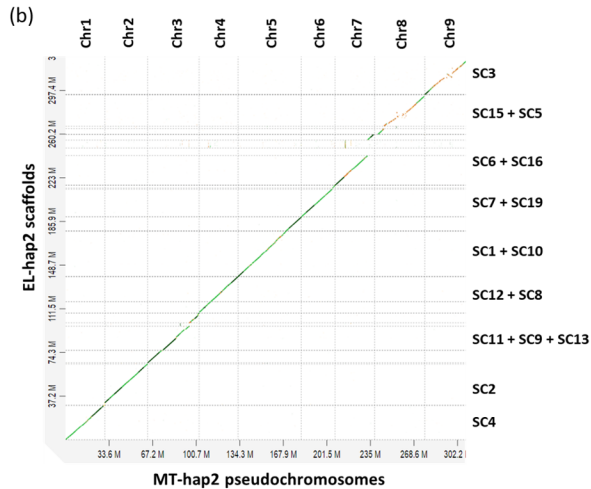

Supplement: Supplementary file 2 — Supplemental Figure S2 The alignment of the scaffolds of Ellendale hap2 assembly against the Murcott haplotypes. [file TPG2-19-e70220-s002.pdf]

Prem-hap1 Chr1

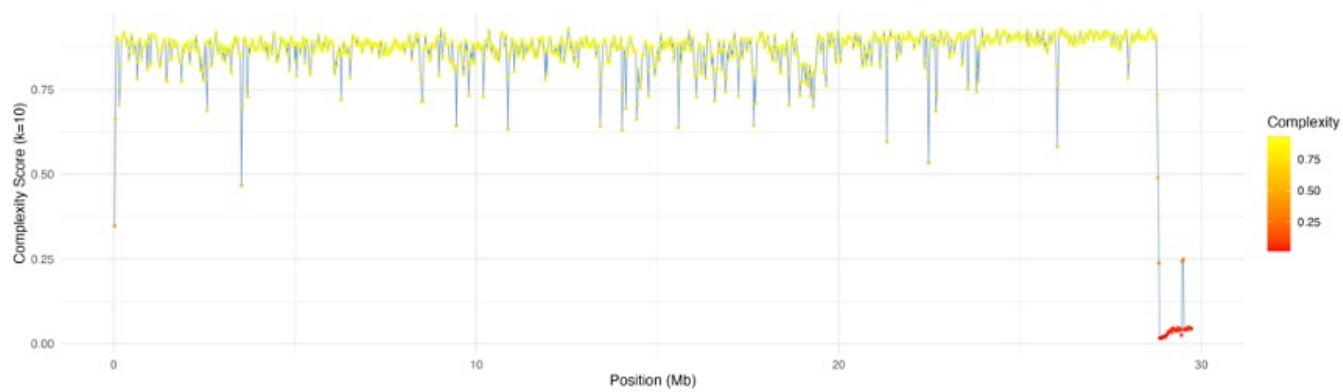

Prem-hap2 Chr1

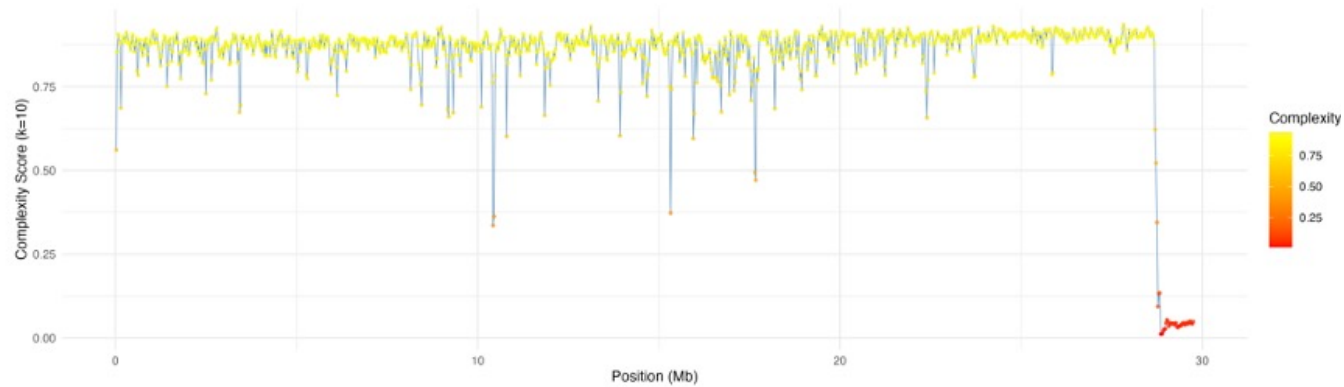

Supplement: Supplementary file 3 — Supplemental Figure S3 Low‐complexity maps of two haplotype assemblies of Citrus Chr1 using distant (k = 10) memory analysis, revealing long‐range sequence patterns. [file TPG2-19-e70220-s005.pdf]

Prem-hap1 Chr1

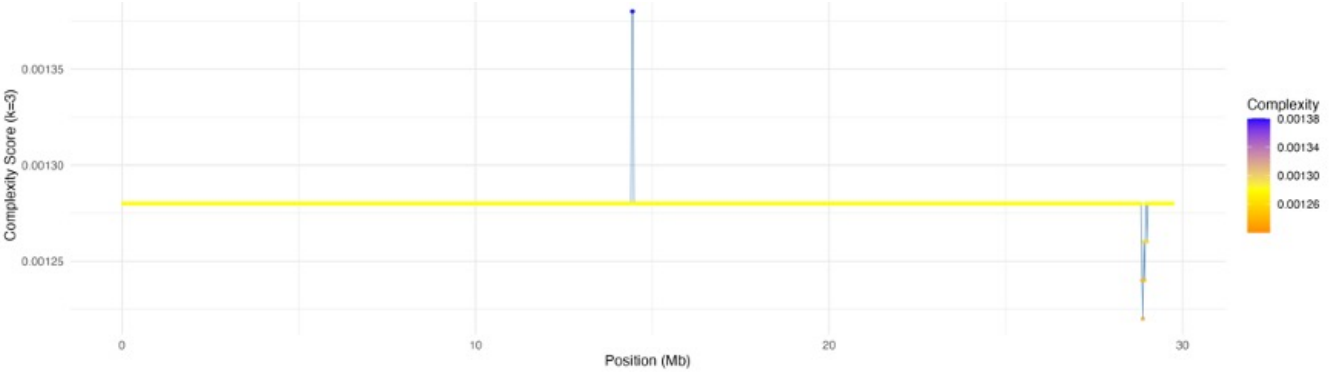

Prem-hap2 Chr1

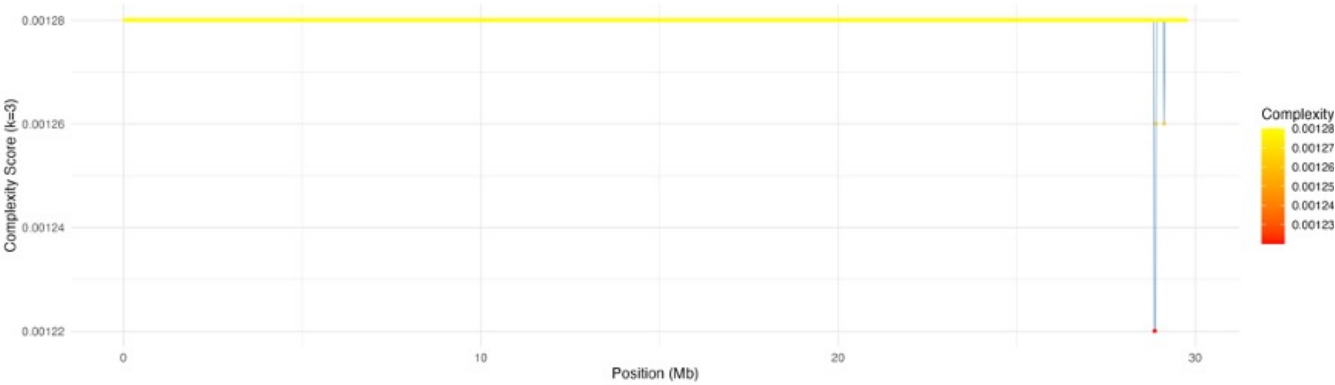

Supplement: Supplementary file 4 — Supplemental Figure S4 Low‐complexity maps of two haplotype assemblies of Citrus Chr1 using local (k = 3) memory analysis, revealing short‐range sequence patterns. [file TPG2-19-e70220-s019.pdf]

Prem-hap1 Chr2

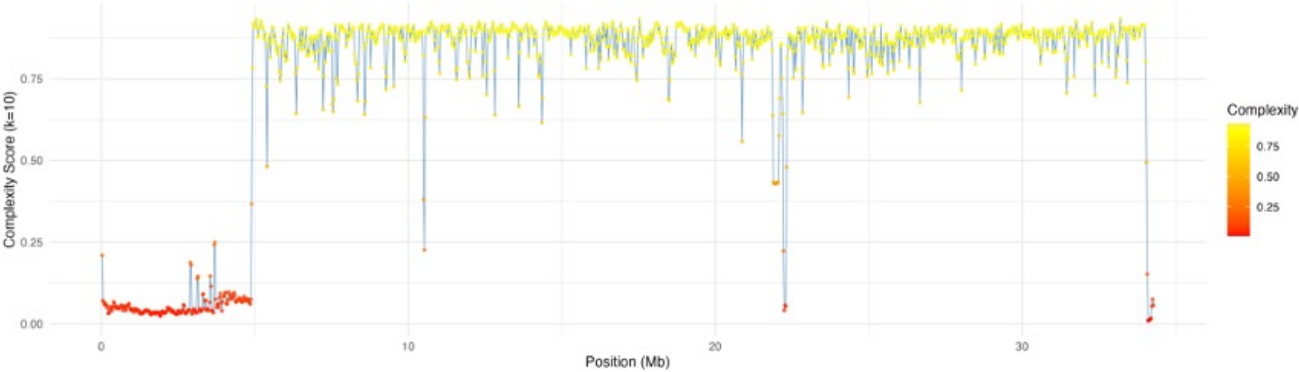

Prem-hap2 Chr2

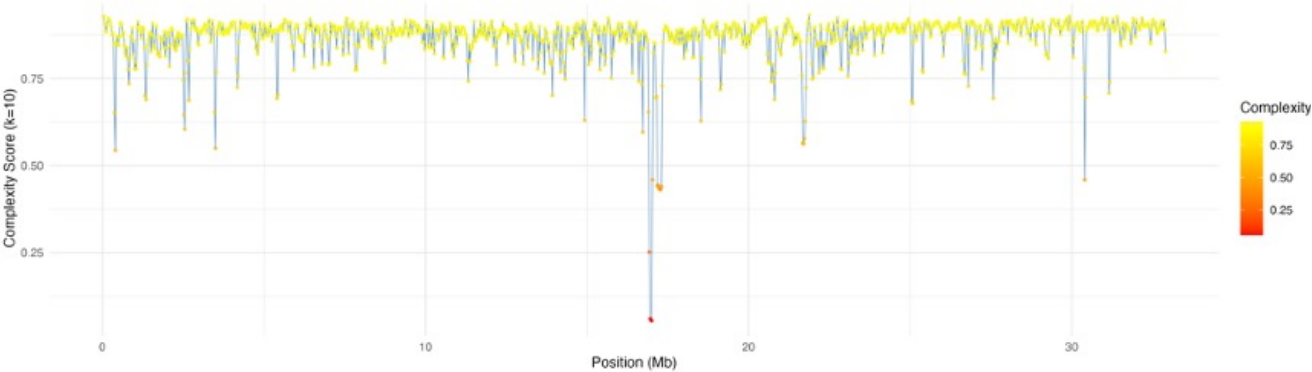

Supplement: Supplementary file 5 — Supplemental Figure S5 Low‐complexity maps of two haplotype assemblies of Citrus Chr2 using distant (k = 10) memory analysis, revealing long‐range sequence patterns [file TPG2-19-e70220-s010.pdf]

Prem-hap1 Chr2

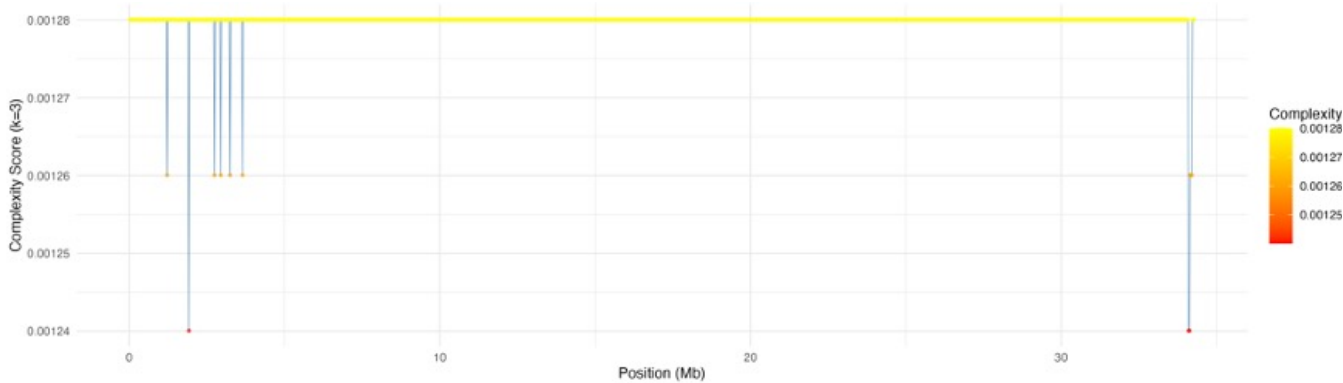

Prem-hap2 Chr2

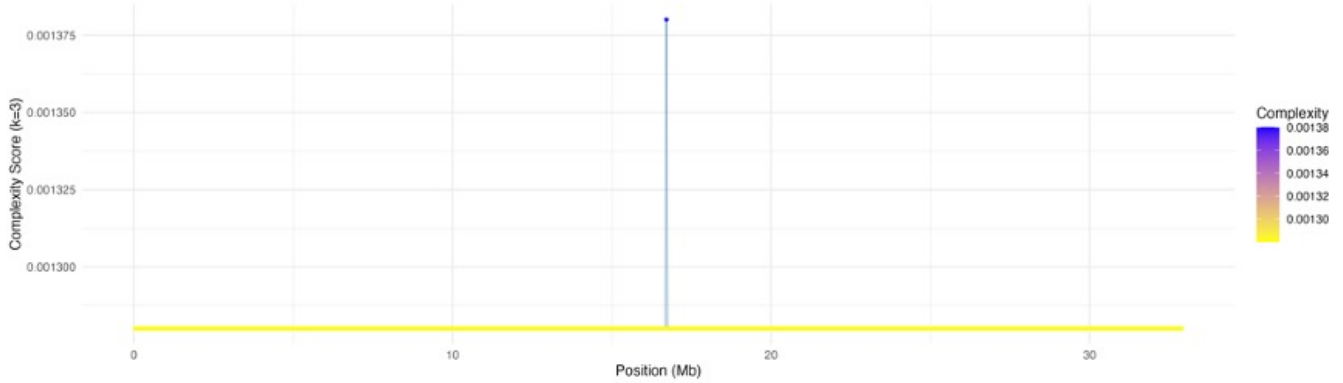

Supplement: Supplementary file 6 — Supplemental Figure S6 Low‐complexity maps of two haplotype assemblies of Citrus Chr2 using local (k = 3) memory analysis, revealing short‐range sequence patterns [file TPG2-19-e70220-s018.pdf]

Prem-hap1 Chr3

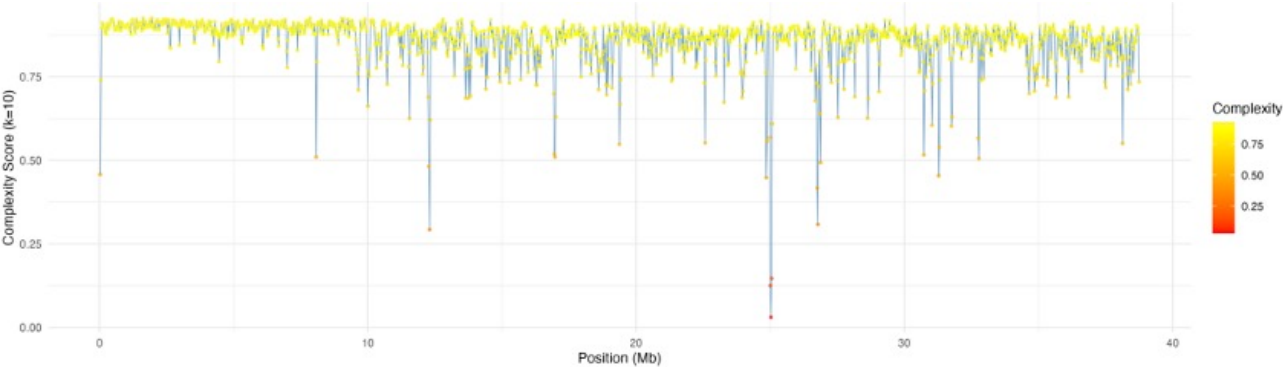

Prem-hap2 Chr3

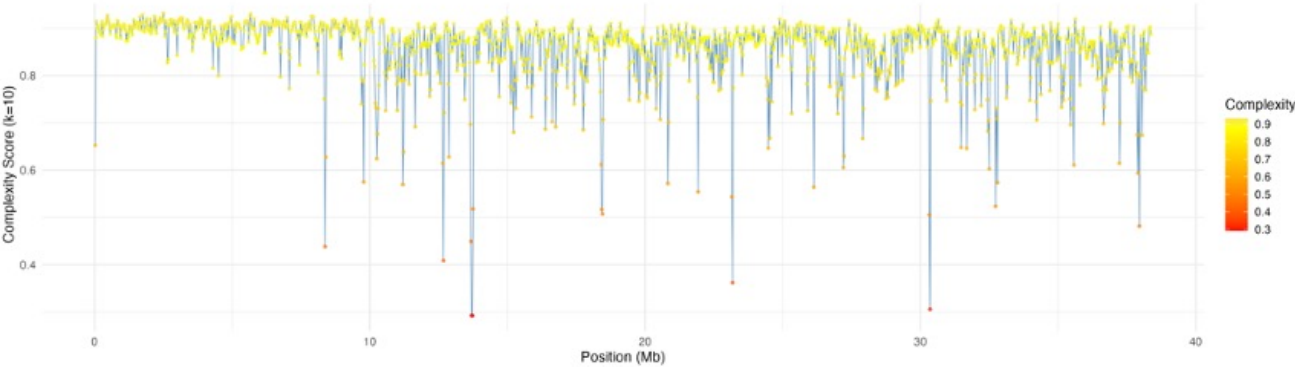

Supplement: Supplementary file 7 — Supplemental Figure S7 Low‐complexity maps of two haplotype assemblies of Citrus Chr3 using distant (k = 10) memory analysis, revealing long‐range sequence patterns [file TPG2-19-e70220-s007.pdf]

Prem-hap1 Chr3

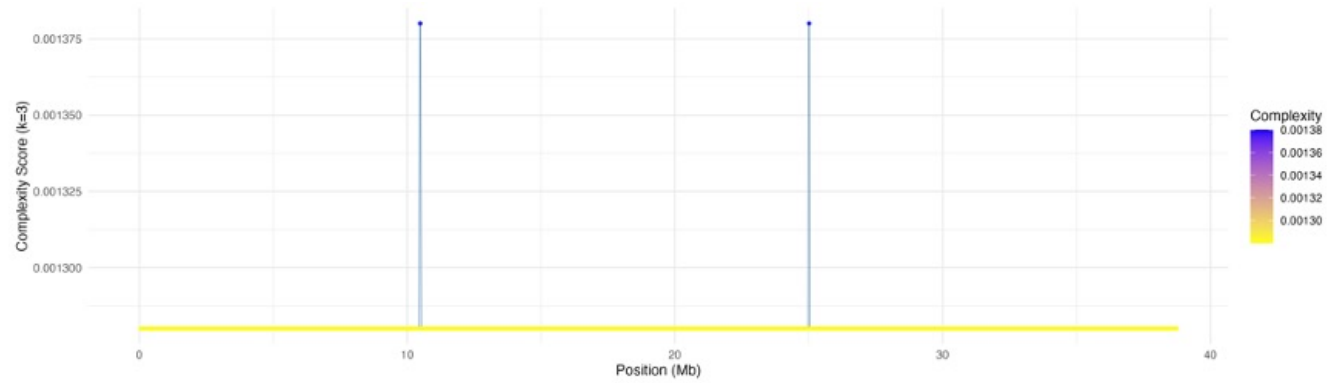

Prem-hap2 Chr3

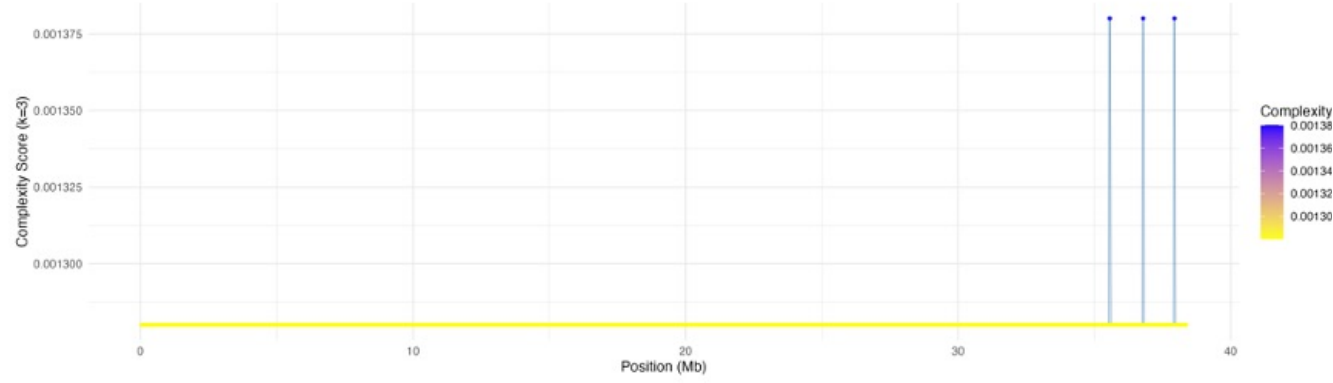

Supplement: Supplementary file 8 — Supplemental Figure S8 Low‐complexity maps of two haplotype assemblies of Citrus Chr3 using local (k = 3) memory analysis, revealing short‐range sequence patterns [file TPG2-19-e70220-s022.pdf]

Prem-hap1 Chr4

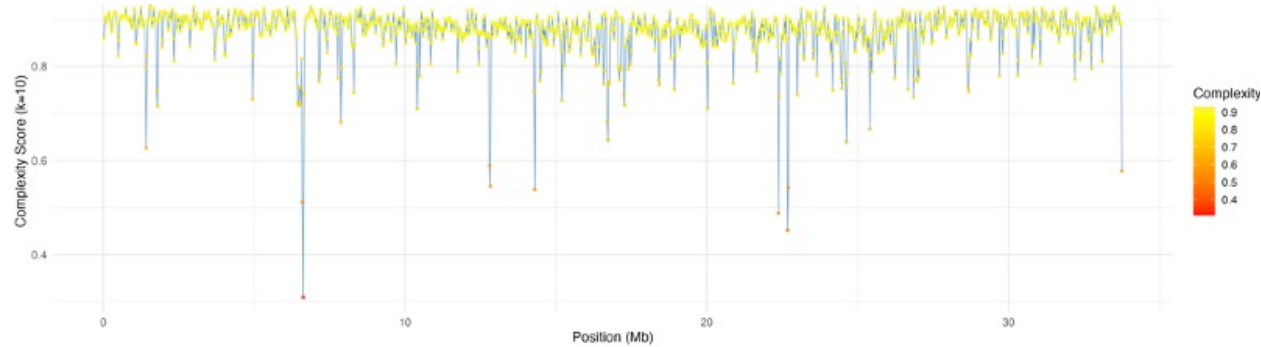

Prem-hap2 Chr4

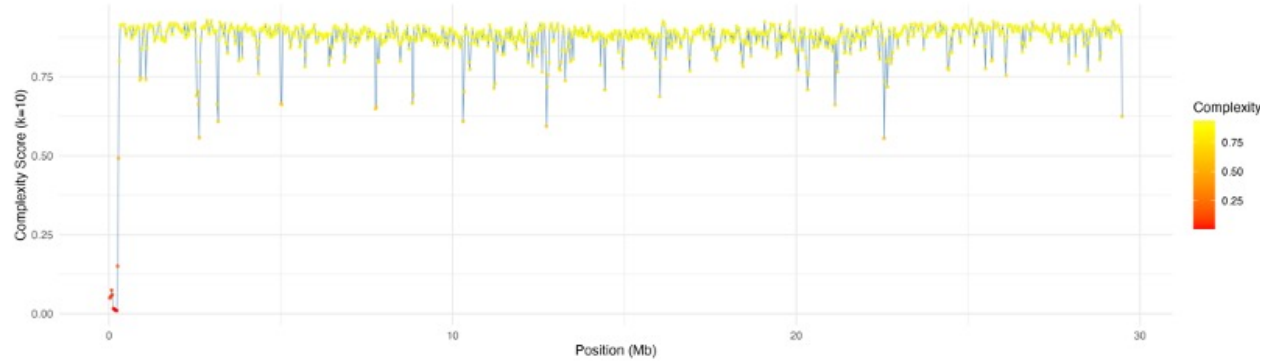

Supplement: Supplementary file 9 — Supplemental Figure S9 Low‐complexity maps of two haplotype assemblies of Citrus Chr4 using distant (k = 10) memory analysis, revealing long‐range sequence patterns [file TPG2-19-e70220-s012.pdf]

Prem-hap1 Chr4

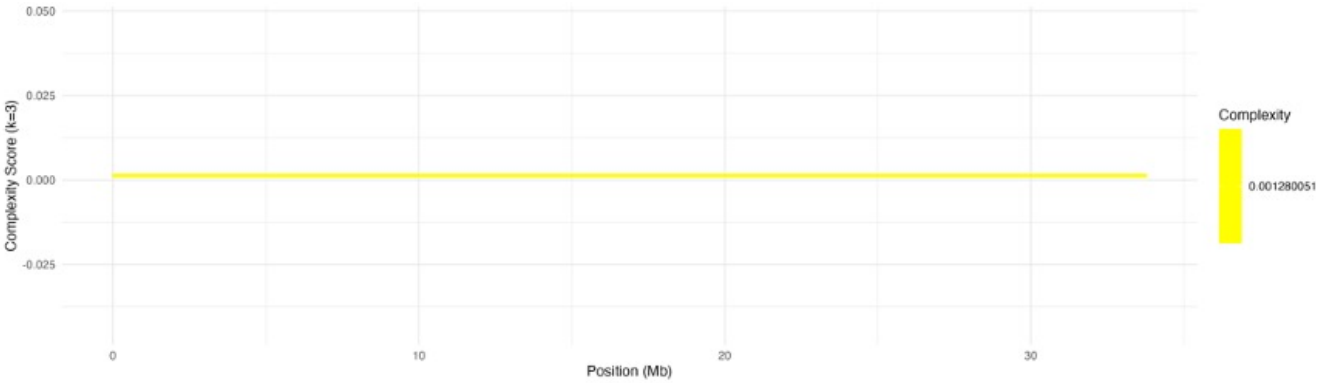

Prem-hap2 Chr4

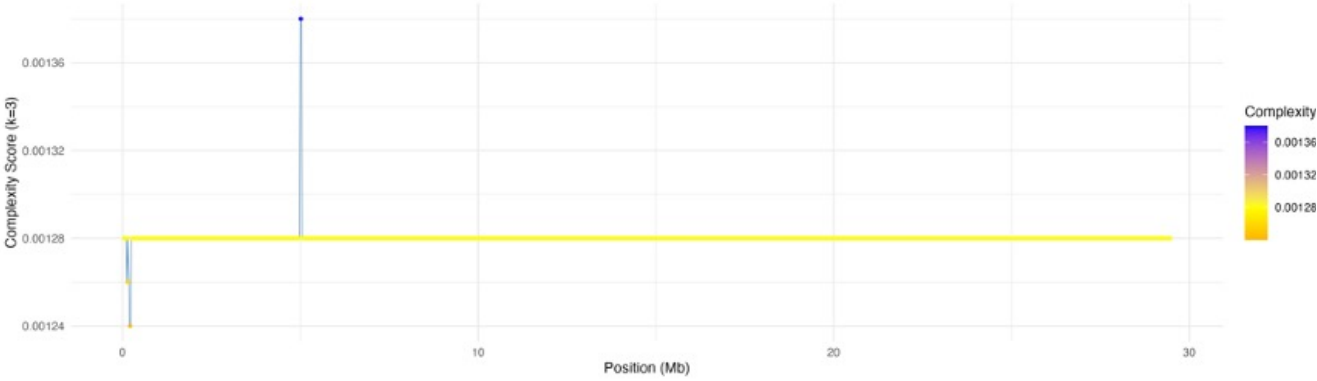

Supplement: Supplementary file 10 — Supplemental Figure S10 Low‐complexity maps of two haplotype assemblies of Citrus Chr4 using local (k = 3) memory analysis, revealing short‐range sequence patterns [file TPG2-19-e70220-s020.pdf]

Prem-hap1 Chr5

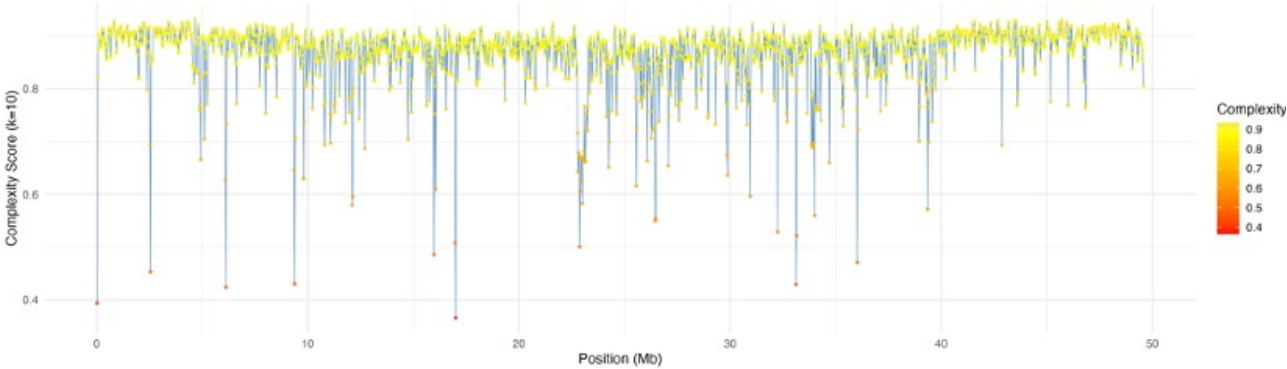

Prem-hap2 Chr5

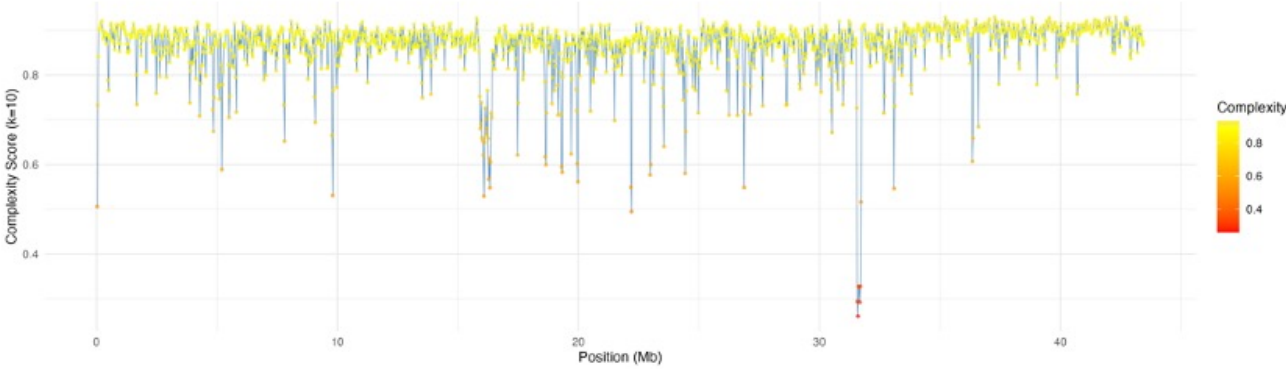

Supplement: Supplementary file 11 — Supplemental Figure S11 Low‐complexity maps of two haplotype assemblies of Citrus Chr5 using distant (k = 10) memory analysis, revealing long‐range sequence patterns [file TPG2-19-e70220-s009.pdf]

Prem-hap1 Chr5

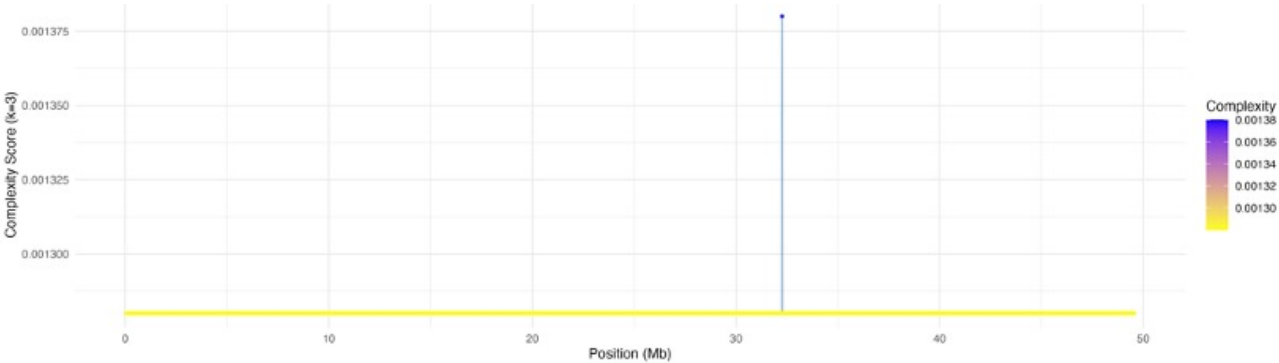

Prem-hap2 Chr5

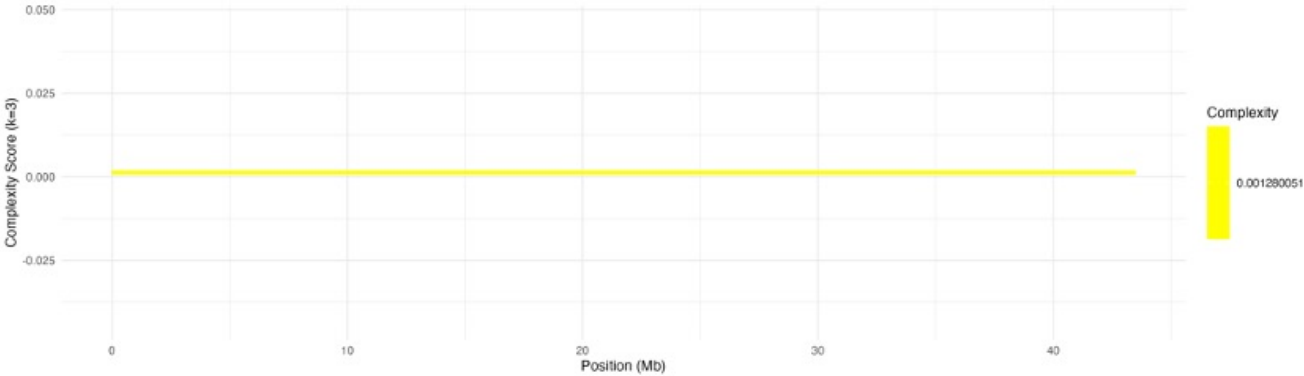

Supplement: Supplementary file 12 — Supplemental Figure S12 Low‐complexity maps of two haplotype assemblies of Citrus Chr5 using local (k = 3) memory analysis, revealing short‐range sequence patterns [file TPG2-19-e70220-s003.pdf]

Prem-hap1 Chr6

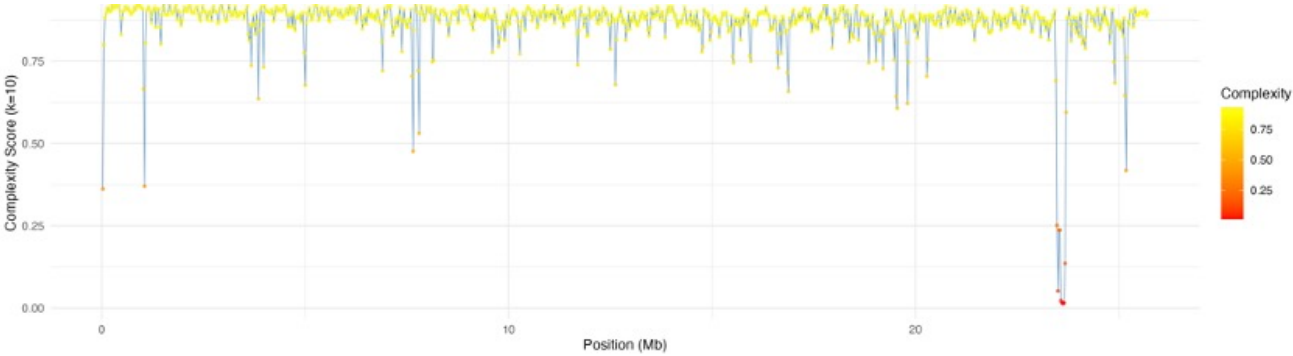

Prem-hap2 Chr6

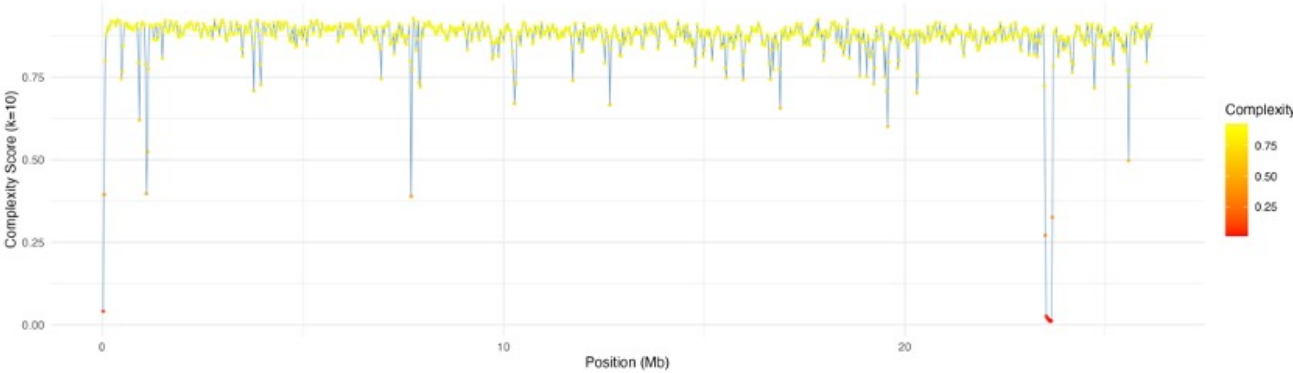

Supplement: Supplementary file 13 — Supplemental Figure S13 Low‐complexity maps of two haplotype assemblies of Citrus Chr6 using distant (k = 10) memory analysis, revealing long‐range sequence patterns [file TPG2-19-e70220-s004.pdf]

Prem-hap1 Chr6

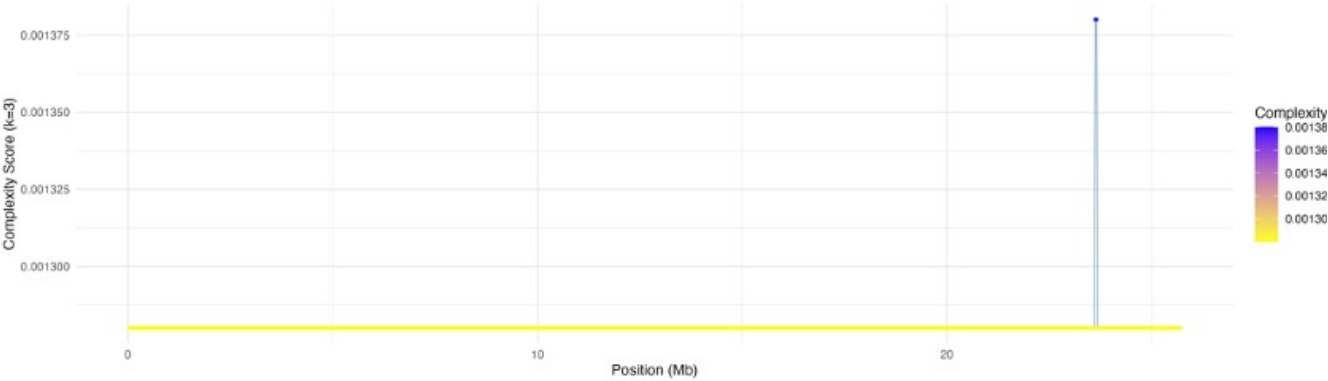

Prem-hap2 Chr6

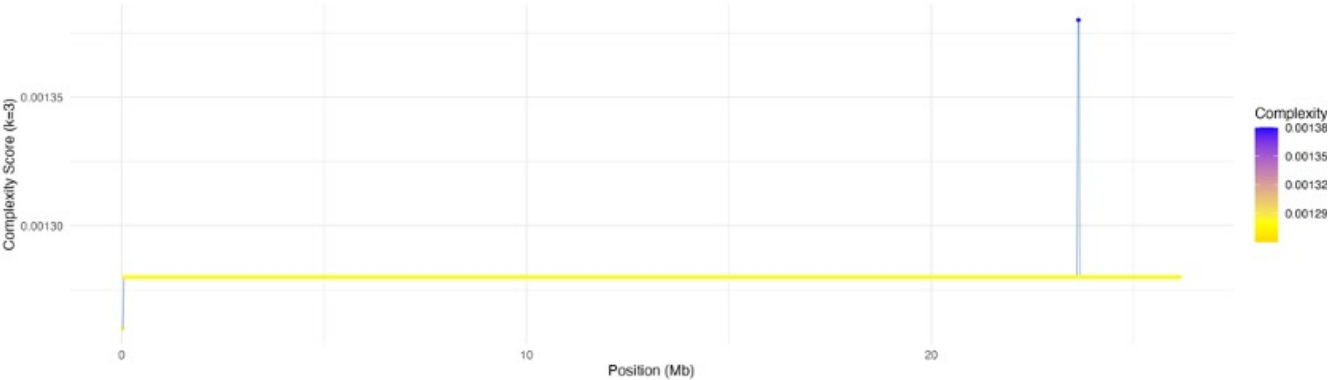

Supplement: Supplementary file 14 — Supplemental Figure S14 Low‐complexity maps of two haplotype assemblies of Citrus Chr6 using local (k = 3) memory analysis, revealing short‐range sequence patterns [file TPG2-19-e70220-s024.pdf]

Prem-hap1 Chr7

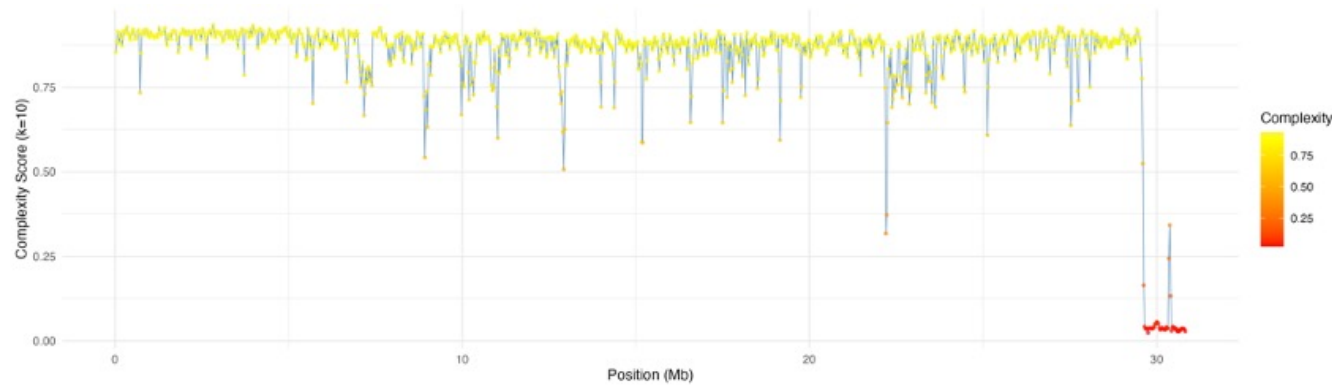

Prem-hap2 Chr7

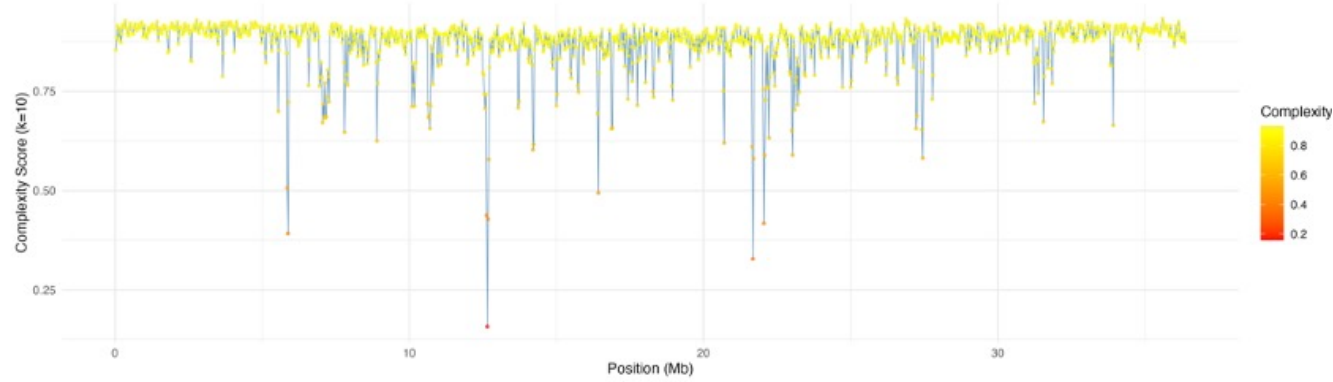

Supplement: Supplementary file 15 — Supplemental Figure S15 Low‐complexity maps of two haplotype assemblies of Citrus Chr7 using distant (k = 10) memory analysis, revealing long‐range sequence patterns [file TPG2-19-e70220-s011.pdf]

Prem-hap1 Chr7

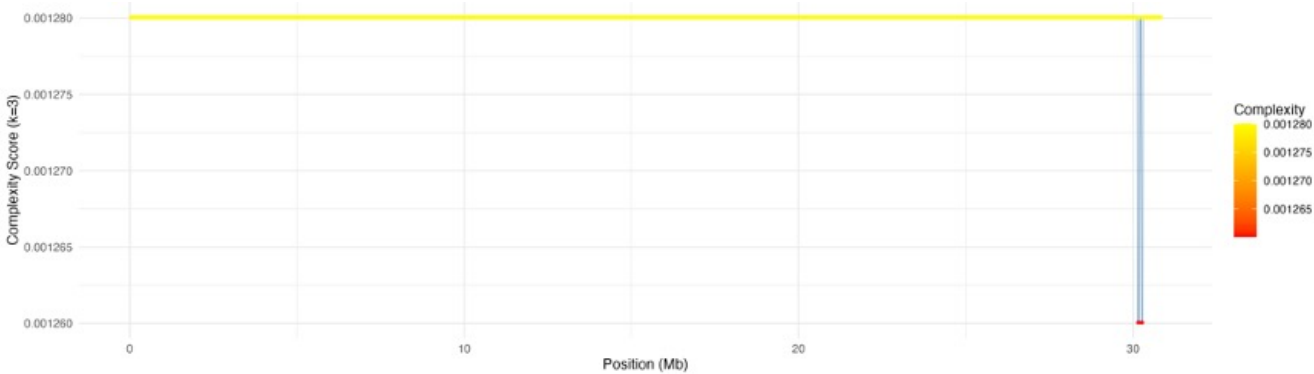

Prem-hap2 Chr7

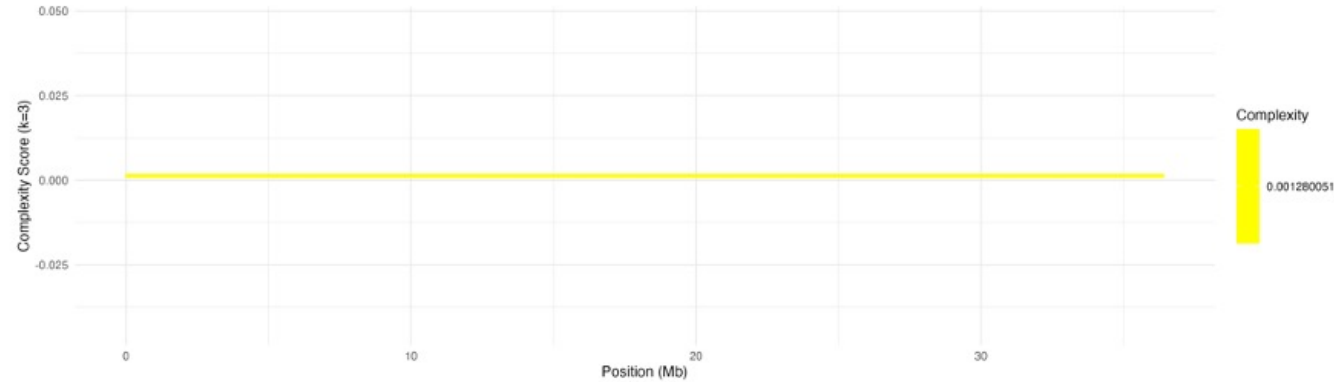

Supplement: Supplementary file 16 — Supplemental Figure S16 Low‐complexity maps of two haplotype assemblies of Citrus Chr7 using local (k = 3) memory analysis, revealing short‐range sequence patterns [file TPG2-19-e70220-s001.pdf]

Prem-hap1 Chr8

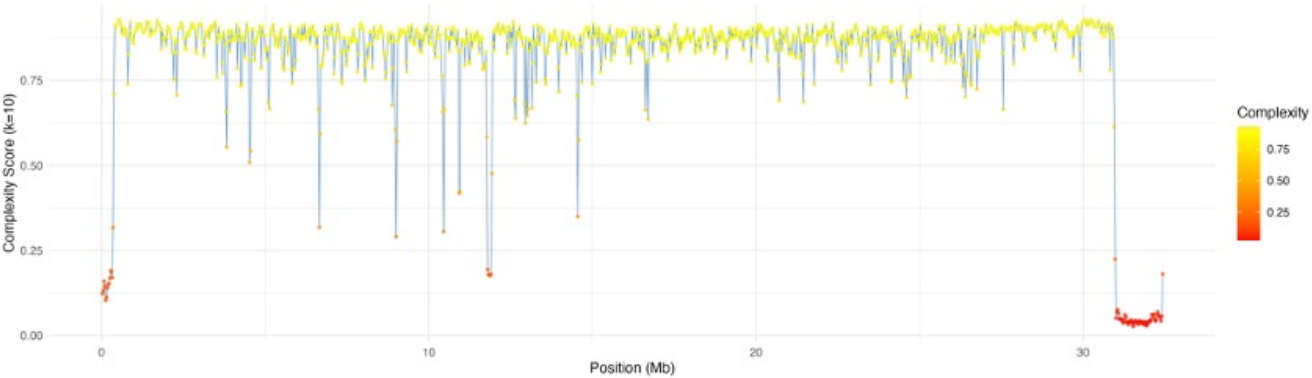

Prem-hap2 Chr8

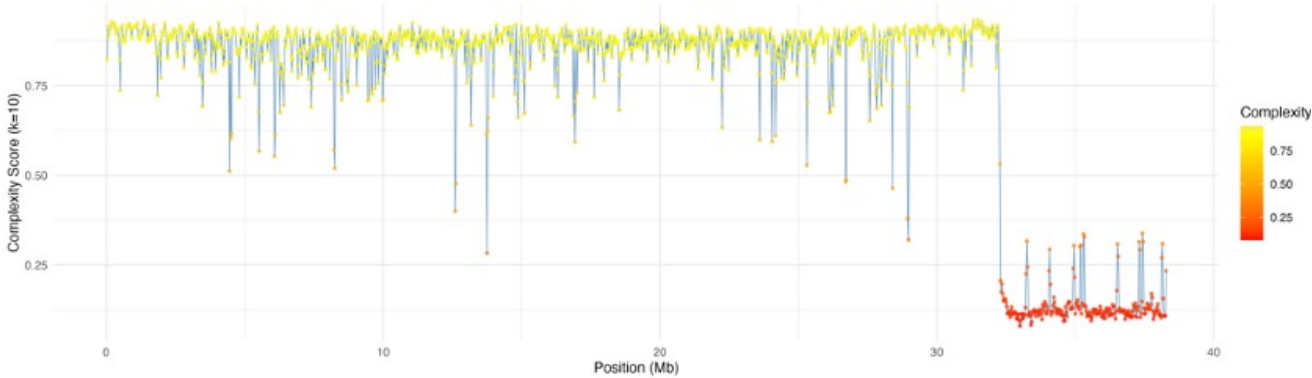

Supplement: Supplementary file 17 — Supplemental Figure S17 Low‐complexity maps of two haplotype assemblies of Citrus Chr8 using distant (k = 10) memory analysis, revealing long‐range sequence patterns [file TPG2-19-e70220-s006.pdf]

Prem-hap1 Chr8

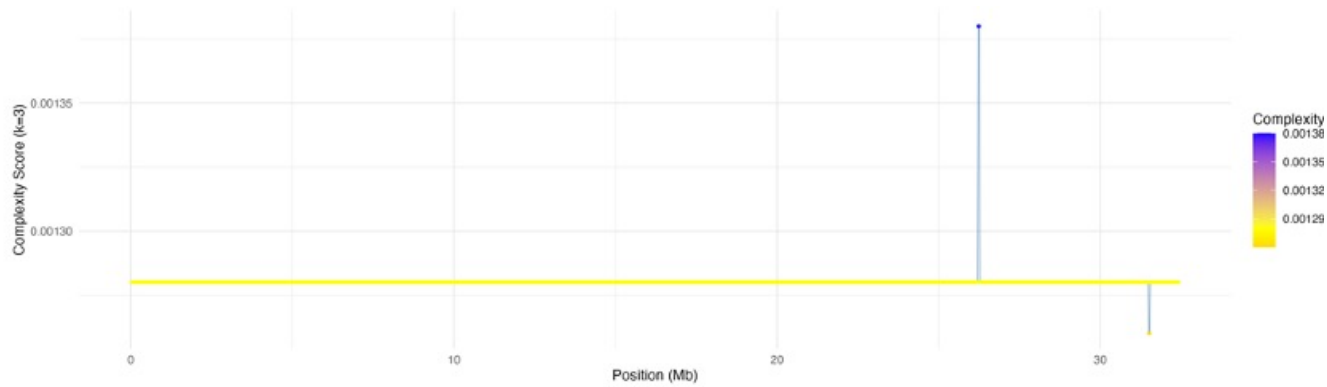

Prem-hap2 Chr8

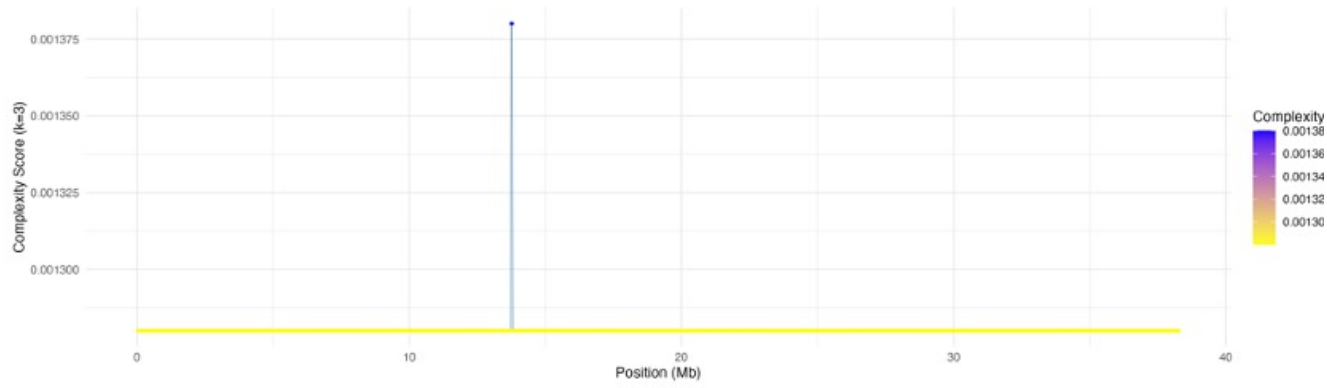

Supplement: Supplementary file 18 — Supplemental Figure S18 Low‐complexity maps of two haplotype assemblies of Citrus Chr8 using local (k = 3) memory analysis, revealing short‐range sequence patterns [file TPG2-19-e70220-s021.pdf]

Prem-hap1 Chr9

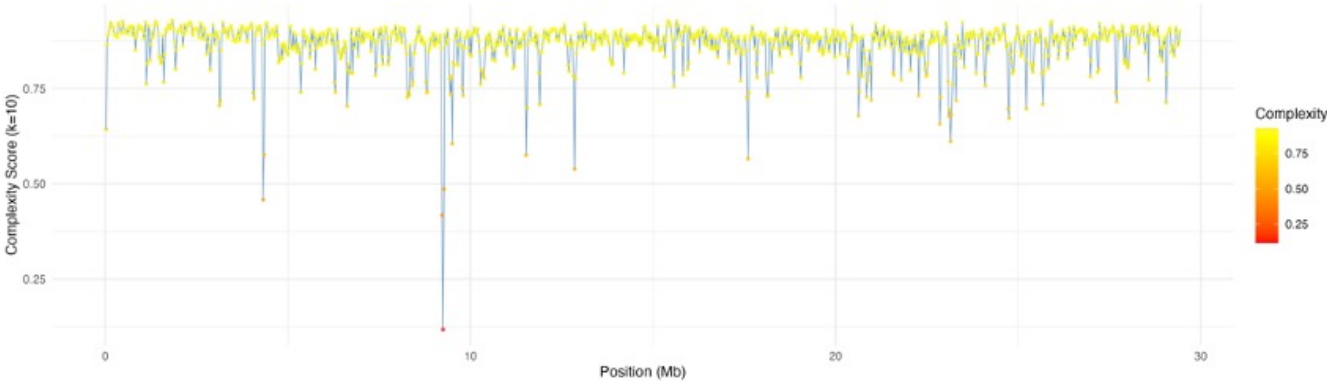

Prem-hap2 Chr9

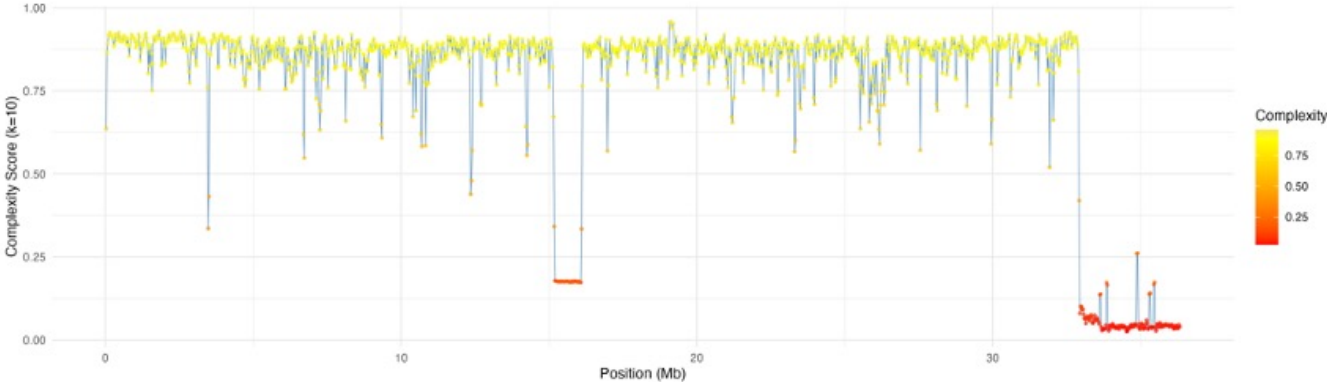

Supplement: Supplementary file 19 — Supplemental Figure S19 Low‐complexity maps of two haplotype assemblies of Citrus Chr9 using distant (k = 10) memory analysis, revealing long‐range sequence patterns [file TPG2-19-e70220-s013.pdf]

Prem-hap1 Chr9

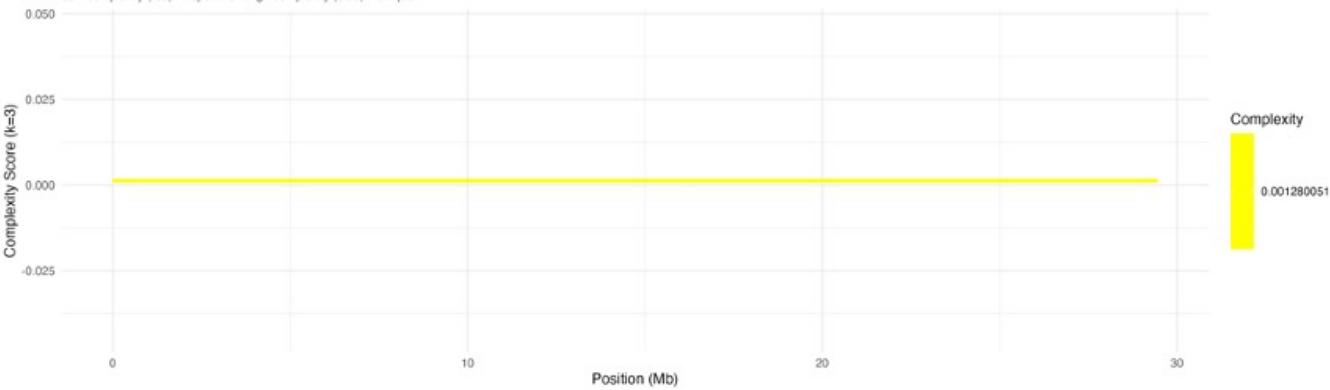

Prem-hap2 Chr9

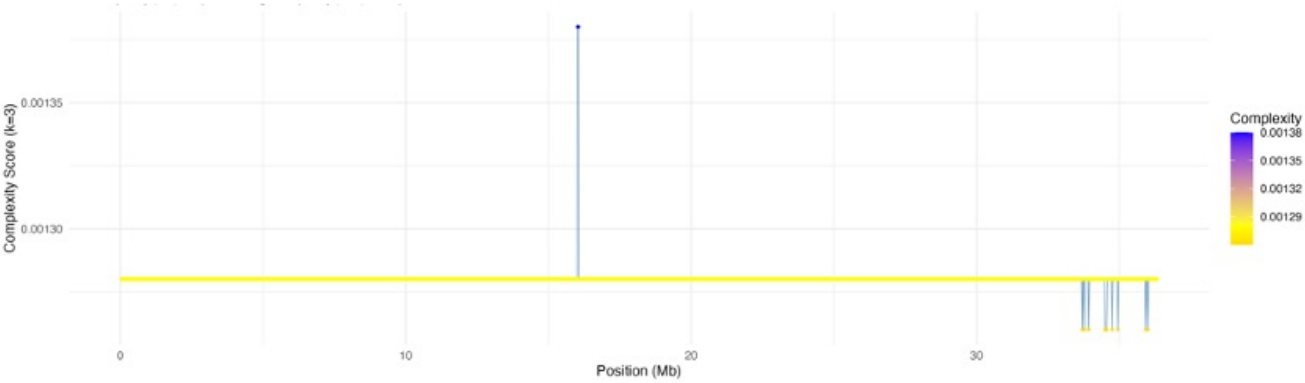

Supplement: Supplementary file 20 — Supplemental Figure S20 Low‐complexity maps of two haplotype assemblies of Citrus Chr9 using local (k = 3) memory analysis, revealing short‐range sequence patterns [file TPG2-19-e70220-s014.pdf]
